# Sex-specific effects of fecal microbiota transplantation on TBI-exacerbated Alzheimer’s disease pathology in mice

**DOI:** 10.3389/fmicb.2025.1703708

**Published:** 2026-02-02

**Authors:** Sirena Soriano, Austin Marshall, Morgan Holcomb, Hannah Flinn, Marissa Burke, Göknur Kara, Paula Scalzo, Sonia Villapol

**Affiliations:** 1 Department of Neurosurgery and Center for Neuroregeneration, Houston Methodist Research Institute, Houston, TX, United States; 2 Department of Computer Science, Rice University, Houston, TX, United States; 3 Department of Neuroscience in Neurological Surgery, Weill Cornell Medical College, New York, NY, United States

**Keywords:** Alzheimer’s disease, fecal microbiota transplantation, gut-brain axis, microbiome, neuroinflammation, sex differences, short-chain fatty acids, traumatic brain injury

## Abstract

**Background:**

Traumatic brain injury (TBI) accelerates Alzheimer’s disease (AD) pathology and neuroinflammation, potentially via gut-brain axis disruptions. Whether restoring gut microbial homeostasis mitigates TBI-exacerbated AD features remains unclear, particularly with respect to sex differences.

**Objective:**

The goal of our study was to test whether fecal microbiota transplantation (FMT) modifies amyloid pathology, neuroinflammation, gut microbial composition, metabolites, and motor outcomes in male and female 5xFAD mice subjected to TBI.

**Methods:**

Male and female 5xFAD mice received sham treatments or controlled cortical impact, followed 24 h later by vehicle (VH) or sex-matched FMT from C57BL/6 donors. Assessments at baseline, 1-, and 3-days post-injury (dpi) included Thioflavin-S and 6E10 immunostaining for Aβ, Iba-1 and GFAP for glial activation, lesion volume, rotarod performance, 16S rRNA sequencing for microbiome profiling, serum short-chain fatty acids (SCFAs), and gut histology.

**Results:**

TBI increased cortical and dentate gyrus Aβ burden, with females showing greater vulnerability. FMT reduced Aβ deposition in sham animals and shifted plaque morphology but did not attenuate TBI-induced amyloid escalation. FMT differentially modulated glial responses by sex and region (reduced microgliosis in males) without altering lesion volume at 3 dpi. Rotarod performance was better in sham females compared to males and declined in FMT-treated TBI females. Fecal microbiome alpha diversity and richness were unchanged, while beta diversity revealed marked, time-dependent community shifts after TBI that were slightly altered by FMT. Gut morphology remained broadly intact, but crypt width increased after TBI, particularly in males.

**Conclusion:**

In 5xFAD mice, TBI drives sex-dependent worsening of amyloid pathology, neuroinflammation, and dysbiosis. Acute FMT partially restores microbial composition and plaque features in sham animals but fails to reverse TBI-induced neuroinflammation or motor deficits. These findings underscore the context- and sex-dependence of microbiome interventions and support longer-term, sex-specific strategies for AD with comorbid TBI.

## 1 Introduction

Alzheimer’s disease (AD) is a progressive and debilitating neurodegenerative condition characterized by memory loss, cognitive decline, and pathological accumulation of amyloid-beta (Aβ) plaques and neurofibrillary tangles in the brain (DeTure and Dickson, 2019). Affecting millions of individuals globally, AD currently lacks effective disease-modifying therapies. A growing body of evidence suggests that the pathogenesis of AD is multifactorial, involving a complex interplay between genetic predisposition, environmental influences, neuroinflammation, and systemic metabolic dysfunction. Among emerging risk factors, traumatic brain injury (TBI) has gained considerable attention for its role in accelerating AD pathology and precipitating cognitive impairment (Livingston et al., 2024). Epidemiological studies further support a severity-dependent relationship, whereby more severe TBI confers a greater risk of subsequent AD development (Guo et al., 2000; Plassman et al., 2000). TBI triggers a cascade of neuropathological events that promote Aβ and tau deposition by disrupting their processing and clearance pathways (Stone et al., 2002; Loane et al., 2009; Wu et al., 2020), thereby creating a pro-AD milieu in vulnerable individuals. Proposed mechanisms linking TBI and AD include axonal injury (Tang-Schomer et al., 2012; Zhao et al., 2023), oxidative stress and mitochondrial dysfunction (Tabassum et al., 2025), blood–brain barrier breakdown (Sivandzade et al., 2020), and a sustained neuroinflammatory response (Ramlackhansingh et al., 2011; Hickman et al., 2018). At the molecular level, TBI-induced neuroinflammation and oxidative stress engage pathways such as NF-κB and NLRP3 inflammasome activation(Heneka et al., 2013), upregulate β- and *γ*-secretase activity (Johnson et al., 2010; Heneka et al., 2015), and dysregulate kinases including GSK3β and CDK5, thereby promoting Aβ overproduction, impairing its clearance, and driving tau hyperphosphorylation (Wang et al., 2007). Rodent TBI models, including controlled cortical impact in amyloidogenic strains, have been instrumental in delineating these mechanisms and their translational relevance for AD (Pasam and Dandekar, 2024). In transgenic AD models such as 5xFAD, experimental TBI has been shown to accelerate plaque deposition in a manner that depends on injury severity and timing, further supporting a mechanistic link between brain trauma and amyloid pathology.

Recent studies have linked TBI to disruptions in the gut microbiota, an ecosystem of trillions of microorganisms that interact with the central nervous system through the gut-brain axis (El Baassiri et al., 2024). In our previous study, we found that within 24 h of TBI, there was a marked shift in microbial composition, particularly among *Lactobacillus* strains (Treangen et al., 2018). Such dysbiosis has been reported in both TBI (Nicholson et al., 2019; Celorrio et al., 2021) and AD models (Harach et al., 2017; Vogt et al., 2017; Heston et al., 2023), where it is thought to exacerbate systemic inflammation and cognitive impairment (Zhan et al., 2018). The gut microbiota regulates the immune system, produces neuroactive metabolites such as short-chain fatty acids (SCFAs), and preserves epithelial barrier integrity (Parada Venegas et al., 2019). In our previous studies, administration of SCFA-producing probiotics after TBI reduced neuropathology (Holcomb et al., 2025), while acute-phase antibiotic treatment eliminated detrimental bacteria and conferred neuroprotection (Flinn et al., 2024). In AD, microbiota disruption has also been linked to increased intestinal permeability (“leaky gut”), elevated circulating proinflammatory cytokines, and heightened microglial activation, all of which contribute to neurodegeneration and cognitive decline (Popescu et al., 2024). These findings highlight modulation of the gut microbiota as a promising therapeutic strategy for neurodegenerative diseases.

Fecal microbiota transplantation (FMT) involves transferring intestinal microbiota from a healthy donor to a recipient with dysbiosis. Originally developed to treat recurrent *Clostridioides difficile* infection, FMT has since shown therapeutic potential in metabolic syndrome, inflammatory bowel disease, and neurodegenerative disorders (Kelly et al., 2016; Vendrik et al., 2020). In preclinical AD models, FMT has been reported to reduce Aβ pathology, improve memory function, and modulate neuroinflammation (Sun et al., 2019). In addition, our previous work demonstrated that young wild-type mice subjected to TBI and subsequently receiving FMT from AD animals exhibited exacerbated neuroinflammation and increased lesion size, indicating that the dysbiotic microbiome of AD donors can worsen post-TBI recovery (Soriano et al., 2022). Consistent with this, a recent controlled cortical impact study using FMT revealed sex-specific differences in post-TBI neuroinflammatory and behavioral outcomes (Pasam and Dandekar, 2023), underscoring the importance of sex as a biological variable in microbiome-targeted interventions after brain injury.

Despite these findings, the effects of FMT on TBI-exacerbated AD pathology, particularly concerning sex-specific responses, remain unclear. To address this gap, we employed the 5xFAD mouse model of AD, which develops early and aggressive Aβ plaque deposition at 5 months old. We subjected these mice to TBI to accelerate neuropathological progression. We hypothesized that FMT from young, wild-type donors could ameliorate TBI-induced gut dysbiosis and neuropathology, thereby restoring microbial homeostasis. In addition, we examined sex as a biological variable influencing treatment response. Our results support that TBI exacerbates neuroinflammatory and neuropathological outcomes in AD mice, with females exhibiting more pronounced pathology. Importantly, although FMT transiently restored aspects of microbial balance, it only reversed the accelerated Aβ accumulation of 5xFAD mice without injury. These findings underscore the role of FMT in driving sex-dependent neurodegenerative processes and highlight the need for more targeted microbiome-based interventions.

## 2 Materials and methods

### 2.1 TBI in an AD mouse model

**Figure 1:**
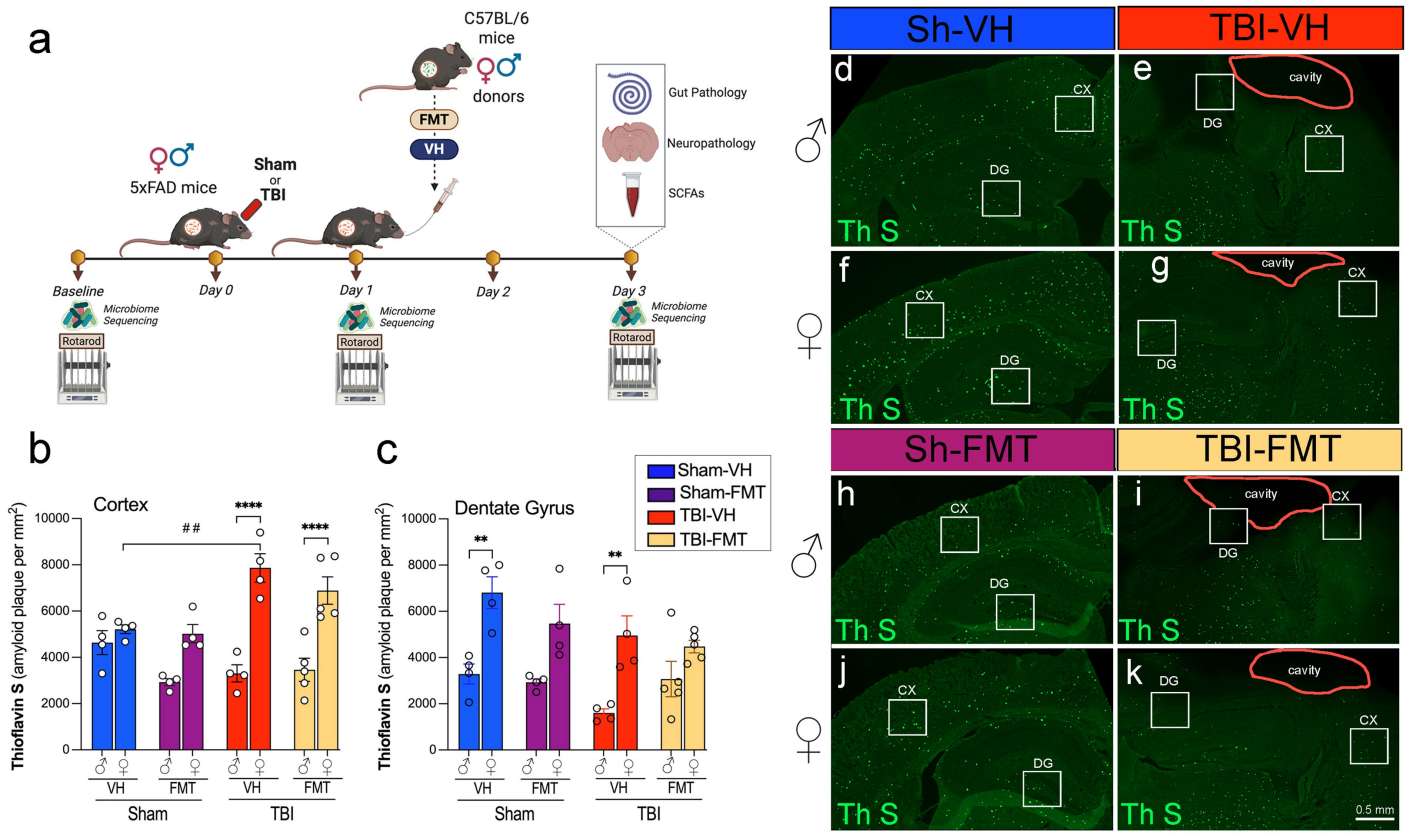
Experimental design and Thioflavin-S–positive amyloid plaques after TBI and FMT. **(a)** Experimental timeline. Male and female 5xFAD mice underwent baseline rotarod testing and fecal collection for microbiome sequencing, then received Sham or TBI surgery (day 0). On day 1, mice were treated with vehicle (VH) or FMT from young sex-matched C57BL/6 donors via oral gavage. Rotarod and microbiome sequencing were repeated on days 1 and 3. On day 3, brains and gut tissue were collected for histology, neuropathology, and SCFA analysis. Quantification of Thioflavin-S-(Th S)-positive plaques in the **(b)** cortex and **(c)** dentate gyrus (DG) of male (♂) and female (♀) mice across Sham-VH, Sham-FMT, TBI-VH, and TBI-FMT groups. **(d–k)** Representative Thioflavin-S staining in coronal sections showing cortex (CX), DG, and lesion cavity (outlined) in male **(d,e,h,i)** and female **(f,g,j,k)** mice. Data are mean ± SEM. Statistics: three-way ANOVA with Bonferroni *post hoc* tests. Symbols indicate significant effects: *sex differences, #Sham vs. TBI. Scale bar: 50 μm.

Young adult (2-month-old) C57BL/6 J mice (Jackson Laboratories, Bar Harbor, ME) and 5xFAD (5-month-old) mice were housed at the Houston Methodist Research Institute animal facilities under a standard 12-h light and dark cycle with ad libitum access to food and water. We used qPCR-based genotyping on tail biopsies to confirm hemizygous and WT genotypes for the 5xFAD strain, using the genotyping service provided by Transnetyx, Inc. (Cordova, TN, USA). All experiments were approved by the Animal Care and Use Committee (IACUC) at Houston Methodist Research Institute, Houston (Texas, USA). Studies were conducted following the NRC guide to the Care and Use of Laboratory Animals. The 5xFAD mice were randomly assigned to eight groups based on sex (male or female), injury type (Sham or TBI), and treatment (vehicle (VH) or FMT). Anesthesia was induced with 4–5% isoflurane and maintained at 1.5–2% with an oxygen flow rate of 1–1.5 L/min during the surgeries. Once anesthetized, each mouse was secured in a stereotaxic frame for the surgical procedure, and controlled cortical impact (CCI) injury was induced on the left hemisphere, targeting the primary motor and somatosensory cortex. This procedure was conducted using an electromagnetic Impact One stereotaxic impactor (Leica Microsystems, Buffalo Grove, IL, USA) positioned 2 mm lateral and 2 mm posterior to Bregma, with an impact depth of 1.5 mm, using a 2-mm diameter flat impact tip at a speed of 3.6 m/s and a dwell time of 100 ms. This TBI model induces an acute and chronic inflammatory response, as shown in our previous work (Villapol et al., 2012; Villapol et al., 2014; Villapol et al., 2015; Villapol et al., 2017). Sham animals underwent all surgical procedures except the cortical impact. Mice were anesthetized and euthanized at 3 days post-injury (dpi), and brains, blood, and intestines were collected for downstream analyses (Figure 1a).

### 2.2 Rotarod test

Sensorimotor function was evaluated using a Rotarod system (Rotamex 5, Columbus Instruments, Columbus, OH) as previously described (Villapol et al., 2012; Villapol et al., 2015). Mice received three training trials 2 days before testing. For each trial, mice were placed on a rotating rod for 30 s, after which the rotation speed increased from 4 to 40 rpm over 5 min. The trial ended when the mouse fell or 5 min elapsed, and the latency to fall was recorded. The mean latency from 3 trials was used as the performance measure at baseline, 1 dpi, and 3 dpi.

### 2.3 Fecal microbiota transplantation

Cecum samples were collected from 2-month-old C57BL/6 male and female mice, which served as donors for FMT. Cecal content from 3 donors of the same sex (3 males and 3 females) was homogenized in 7.5 mL of sterile phosphate-buffered saline (PBS) and centrifuged at 800*g* for 3 min to pellet the large particles. The supernatants were collected in fresh sterile tubes, and the final bacterial suspension was filtered through a 70 μm strainer, collected in fresh sterile tubes, and stored at −80 °C. A single 200 μL bolus of the prepared microbiota suspension was administered by oral gavage to each recipient 5xFAD mouse at 24 h after TBI (Figure 1a), by matching the sex of the FMT recipients and donors. Mice from the VH group received an equal volume of sterile PBS as the control group.

### 2.4 Fecal microbiome DNA extraction

Fresh stool pellets were aseptically collected and placed in sterile tubes, immediately snap-frozen, and subsequently stored at −80 °C for preservation. Genomic bacterial DNA was extracted from the frozen stool samples utilizing the QIAamp PowerFecal Pro DNA Kit (Qiagen, Germantown, MD). To facilitate DNA extraction, bead beating was conducted in three cycles, each lasting 1 min, at a speed of 6.5 m/s. There was a 5-min rest period between each cycle. This mechanical disruption was performed using a FastPrep-24 system (MP Biomedicals, Irvine, CA). Following the bead-beating process, the DNA isolation proceeded per the manufacturer’s instructions. The concentration of the extracted genomic DNA was subsequently quantified using a DS-11 Series Spectrophotometer/Fluorometer (DeNovix, Wilmington, DE).

### 2.5 Sequencing of 16S rRNA V1-V3 regions

The V1-V3 16S ribosomal RNA gene region was targeted for mouse gut microbiome characterization. The primers used for amplification contain adapters for MiSeq sequencing and single-index barcodes so that the PCR products may be pooled and sequenced directly, targeting at least 10,000 reads per sample (Caporaso et al., 2012). Primers used for the 16S V1-V3 amplification were 27F (AGAGTTTGATYMTGGCTCAG, where Y = C (90%) or T (10%); M = A (30%) or C (70%)) and 534R (ATTACCGCGGCKGCTGG, where K = G (10%) or T (90%)) (Weisburg et al., 1991). Sequencing libraries for the V1-V3 target were constructed following the instructions provided by the Illumina MiSeq system, with end products of 300 bp paired-end libraries.

### 2.6 Amplicon sequence analysis pipeline

Raw data files in binary base call (BCL) format were converted and demultiplexed into FASTQs based on the single-index barcodes using the Illumina ‘bcl2fastq’ software. Forward and reverse read pairs underwent quality filtering using bbduk.sh (BBMap version 38.82), removing Illumina adapters, PhiX reads, and sequences with a Phred quality score below 15 and length below 100 bp after trimming. Quality-controlled 16S V1-V3 reads were then merged using bbmerge.sh (BBMap version 38.82), with parameters optimized for the V1-V3 amplicon (vstrict = t qtrim = t trimq = 15). Further processing was performed using custom R and bash scripts, which can be found in our documentation at https://github.com/villapollab/fmt_ad_no_abx. Sequences were processed sample-wise (independent) with DADA2 v1.36 to remove PhiX contamination, trim reads (forward reads at 275 bp and reverse reads at 265 bp; reads shorter were discarded), discard reads with > 2 expected errors, correct errors, merge read pairs, and to remove PCR chimeras. After clustering, 1,839 amplicon sequencing variants (ASVs) were obtained across all samples. The ASV count table contained a total of 1,036,836 counts, at least 4,115 and at most 16,686 per sample (average 10,266). Taxonomic classification was performed in DADA2 with the assigned taxonomy function using the precompiled GreenGenes2 release 2024.09 databases (McDonald et al., 2024). The DADA2 ASV matrix, taxonomy table, and the study metadata table were combined for use within a phyloseq v1.52.0 object and merged with the phylogenetic tree externally calculated from the ASVs using MAFFT v7.525 alignment and FastTree v2.1.11 phylogenetic tree construction. The phyloseq object was used to calculate alpha and beta diversity indices, PERMANOVA calculations with vegan⸬adonis2 v2.6–10, relative abundance bar plots with microViz v0.12.6, R v4.5.0, and differentially abundant taxa calculations using ANCOMBC2 v2.10.1 (Lin and Peddada, 2024).

### 2.7 Serum SCFA analysis

SCFAs were analyzed at 3 dpi by a derivatization procedure. 40 μL of collected blood serum was added to 40 μL of acetonitrile, vortexed, and centrifuged. 40 μL of the supernatant, 20 μL of 200 mM 12C6-3-Nitrophenylhydrazine (3NPH), and 120 mM 1-Ethyl-3-(3-dimethyl aminopropyl) carbodiimide (EDC) were combined. 20 μL of hydrochloric acid was added and incubated for 30 min at 40 °C. The resulting mixture was cooled and made up to 1.91 mL with 10% aqueous acetonitrile. 5 μL of the sample was injected into LC/MS/MS. SCFAs were separated using mobile phases: 0.1% formic acid in water (mobile phase A) and 0.1% formic acid in acetonitrile (mobile phase B). Separation of metabolites was performed on Acquity UPLC HSS T3 1.8 µm (2.1 × 100 mM). The SCFA were measured in ESI negative mode using a 6,495 triple quadrupole mass spectrometer (Agilent Technologies, Santa Clara, CA) coupled to an HPLC system (Agilent Technologies, Santa Clara, CA) with multiple reaction monitoring (MRM). The acquired data was analyzed using Agilent Mass Hunter quantitative software (Agilent Technologies, Santa Clara, CA).

### 2.8 Immunofluorescence analysis

Serial free-floating coronal brain sections, 16 μm thick, were prepared at the level of the dorsal hippocampus for immunohistochemical analysis. The sections were processed using immunohistochemistry protocols, which included three consecutive 5 min washes in PBS containing 0.5% Triton X-100 (PBS-T). Sections were then treated with 5% normal goat serum (NGS) in PBS-T to block nonspecific binding for 1 h at room temperature. Overnight incubation at 4 °C followed, using 3% NGS in PBS-T with primary antibodies targeting anti-rabbit Iba-1 (Wako, CAT#019–19,741, 1:500), anti-mouse Aβ6E10 (BioLegend, CAT#803001, 1:500), and anti-mouse GFAP (Millipore, CAT#MAB360, 1:500). Primary antibodies were chosen based on prior validation in 5xFAD and TBI models and manufacturer-provided specificity data, and staining patterns were confirmed to match published distributions. The following day, the sections were washed 3 times for 5 min each in PBS-T and incubated with the corresponding secondary antibodies (all 1:1000, Invitrogen), for 2 h at room temperature. The sections were then rinsed with PBS three times for 5 min each and incubated in PBS with DAPI solution (1:50,000, Sigma-Aldrich, St. Louis, MO) to counterstain nuclei. The sections were rinsed with distilled water and covered with Fluoro-Gel with Tris Buffer mounting medium (Electron Microscopy Sciences, Hatfield, PA). Quantitative analysis of immunolabeled sections was performed using unbiased standardized sampling techniques across an average of three coronal sections per animal, centered on the lesion epicenter. Analyses focused on the primary somatosensory cortex and hippocampal dentate gyrus, with n = 4–5 per group. Image analysis of the staining in the cortical regions was performed using ImageJ software, as previously described (Villapol et al., 2017).

### 2.9 Thioflavin-S staining

Coronal brain sections (16 μm thick) spanning the hippocampus and cortex were mounted onto gelatin-coated glass slides (SuperFrost Plus, Thermo Fisher Scientific, IL) and air-dried. Slides then underwent a graded ethanol rehydration series (100, 95, 70, and 50%) followed by two washes in distilled water (3 min each). Sections were incubated in 1% aqueous Thioflavin-S solution (Sigma-Aldrich, St. Louis, MO) for 8 min in the dark and subsequently differentiated by two washes in 80% ethanol and one wash in 95% ethanol (3 min each). After differentiation, sections were rinsed in three exchanges of distilled water and coverslipped with Fluoro-Gel with Tris Buffer mounting medium (Electron Microscopy Sciences, Hatfield, PA) to preserve fluorescence. Thioflavin-S-positive amyloid plaques were visualized using an epifluorescence microscope (excitation 450–490 nm, emission 515–565 nm). For quantification, images were acquired from 3 to 4 sections per animal at matched rostro-caudal levels and analyzed using ImageJ software. Amyloid burden was expressed as the percentage area occupied by Thioflavin-S-positive deposits within defined regions of interest (ROI). Quantitative image analysis was performed on thresholded images using standardized macros in ImageJ by an experimenter blinded to group, and signal was expressed as percent immunoreactive area (or plaque count) within anatomically defined ROIs.

### 2.10 Lesion volume

Brain sections were stained with Cresyl-violet. An average of 10–12 coronal brain sections, evenly spaced between 0 and −2.70 mm relative to bregma, were selected for cresyl violet staining to visualize injury-associated regions. Sections were mounted onto gelatin-coated glass slides (SuperFrost Plus, Thermo Fisher Scientific, IL) and immersed in a 1% cresyl violet solution (Sigma-Aldrich, St. Louis, MO), freshly prepared in distilled water and filtered before use. After staining, slides were sequentially dehydrated in graded ethanol solutions (50, 70, 95, and 100%; 2 min each), cleared in xylene (2 × 2 min), and coverslipped using Permount mounting medium (Thermo Fisher Scientific) for long-term preservation. Lesion volume was obtained by multiplying the sum of the lesion areas by the distance between sections. The percent of lesion volume was calculated by dividing each lesion volume by the total ipsilateral hemisphere volume (similarly obtained by multiplying the sum of the areas of the ipsilateral hemispheres by the distance between sections).

### 2.11 Gut histopathological analyses

The small intestines were isolated, combined by experimental group, and fixed O/N in modified Bouin’s fixative (50% ethanol, 5% acetic acid in distilled water). Then, the ilia were prepared using the Swiss-rolling technique and kept in 4% paraformaldehyde until further processing. Tissue samples were processed using a Shandon Excelsior ES Tissue Processor and embedded in paraffin with a Shandon HistoCenter Embedding System, following the manufacturer’s standard protocols. The samples were sectioned at a thickness of 5 μm and mounted onto glass slides. Hematoxylin and Eosin (H&E) staining was performed to assess tissue structure. Intestinal sections were deparaffinized in xylene, rehydrated in water, and stained with hematoxylin for 6 h at 60–70 °C. After rinsing with tap water to remove excess stain, the sections were differentiated using 0.3% acid alcohol for 2 min, followed by eosin staining for 2 min. The slides were then rinsed and mounted with a xylene-based Permount mounting medium, allowing them to dry overnight. Alcian Blue staining was conducted to evaluate mucin production by goblet cells in the intestines. Deparaffinized sections were dehydrated in graded ethanol solutions and washed in distilled water before applying the Alcian Blue solution for 30 min. Excess stain was removed with tap water, followed by counterstaining with Nuclear Fast Red Solution for 5 min. Samples were then rinsed, dehydrated, and cleared in xylene.

### 2.12 Statistical analysis

To evaluate the combined effects of sex, injury status (sham vs. TBI), and treatment (VH vs. FMT), three-way ANOVAs were conducted with Bonferroni *post hoc* testing on pairwise comparisons differing by a single factor. For post-TBI comparisons, two-way ANOVAs were performed to assess the effects of sex and treatment, followed by Bonferroni’s multiple comparisons post hoc test. Non-parametric Kruskal-Wallis tests were used for microbiota diversity data. All mice were randomly assigned to experimental conditions, and experimenters were blinded to the treatment groups throughout the study. Statistical analyses were conducted using GraphPad Prism 9 software. (GraphPad, San Diego, CA, USA). Data are presented as mean ± standard error of the mean (SEM), and significance levels were set at *p < 0.05, **p < 0.01, ***p < 0.001. Symbols in figures denote significance as follows: (*) indicates sex differences, (#) indicates differences between sham and TBI groups, and (^★^) denotes treatment effects in the ANOVAs post-hoc tests with Bonferroni correction.

## 3 Results

### 3.1 Sex-specific effects on amyloid deposition in the cortex and dentate gyrus post-TBI

To investigate the impact of FMT on amyloid pathology following TBI, we used 5xFAD mice subjected to sham or TBI surgery and subsequently transplanted with VH or microbiota from C57BL/6 donor mice (Figure 1a). Microbiome sequencing and motor testing were conducted at baseline, 1 dpi and 3 dpi, and tissue analysis was performed at endpoint (3 dpi).

Thioflavin-S (Thio-S) staining revealed abundant amyloid plaque deposition in both the cortex and dentate gyrus (DG) of 5xFAD mice (Figures 1b–k). Quantification showed that cortical plaque burden was significantly increased in female TBI mice compared to female sham controls (##*p* < 0.01), exhibiting higher amyloid burden after injury (Figure 1g). At 3 dpi, female TBI mice exhibited a significantly greater cortical amyloid plaque burden compared to their male counterparts treated with either VH or FMT (****p < 0.0001). FMT did not alter the cortical amyloid burden substantially relative to VH controls. In the DG, amyloid plaque burden was similarly elevated in female VH mice compared to male VH controls under both sham and TBI conditions (Figures 1c−g). However, this difference was not seen in the FMT groups. Together, these data indicate that the amyloid accumulation in 5xFAD mice occurs in a region- and sex-specific manner, and that TBI synergizes with microbial transfer to exacerbate cortical plaque pathology.

### 3.2 FMT modulates Aβ pathology in a sex- and brain region-specific manner

**Figure 2:**
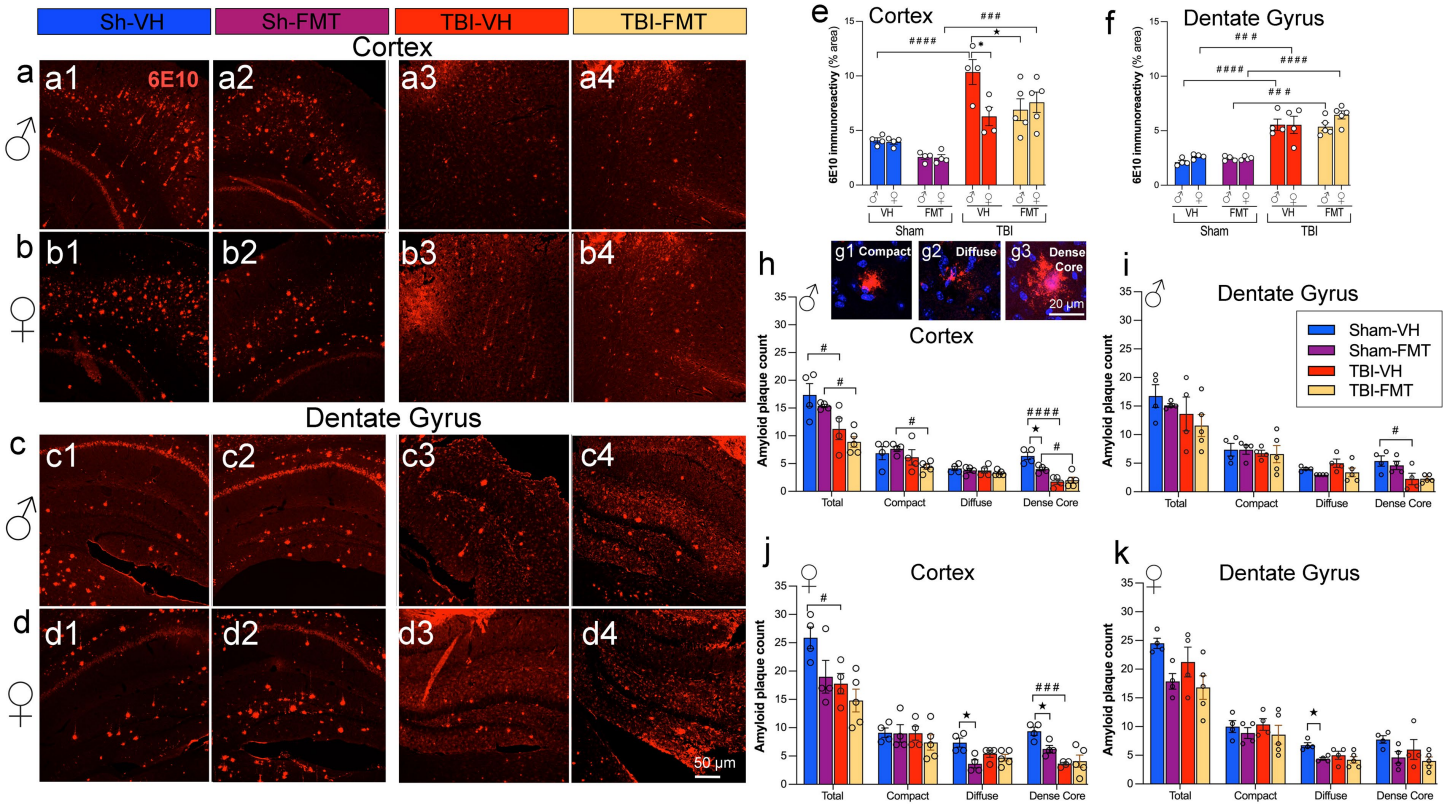
FMT modulates Aβ burden and plaque morphology after TBI. **(a–d)** Representative 6E10 immunostaining in cortex **(a,b)** and dentate gyrus **(c,d)** of male **(a,c)** and female **(b,d)** 5xFAD mice in Sham-VH, Sham-FMT, TBI-VH, and TBI-FMT groups. **(e,f)** Quantification of 6E10 immunoreactivity (% area) in cortex **(e)** and dentate gyrus **(f)** shows increased Aβ burden after TBI in both sexes, with a reduction in cortical 6E10 signal in TBI-FMT males compared with TBI-VH males. **(g1–g3)** Representative examples of compact, diffuse, and dense-core plaques used for morphological classification. **(h,i)** Plaque counts in male cortex **(h)** and dentate gyrus **(i)** stratified by plaque type (total, compact, diffuse, dense-core). **(j,k)** Plaque counts in female cortex **(j)** and dentate gyrus **(k)**. Overall, TBI shifted plaques toward a more diffuse phenotype with a relative reduction in dense-core plaques in both VH- and FMT-treated mice. Data are mean ± SEM. Statistics: three-way ANOVA with Bonferroni correction for 6E10 immunoreactivity **(e,f)**, two-way ANOVA (by sex and plaque type) with Bonferroni correction for plaque counts **(h–k)**. Symbols indicate significant effects: *sex differences, #Sham vs. TBI, ★treatment (VH vs. FMT). Scale bars: 50 μm **(a–d)**, 20 m **(g1–g3)**.

To further evaluate how FMT influences Aβ pathology following TBI, we performed immunostaining with the 6E10 antibody in cortical and hippocampal regions of 5xFAD mice (Figures 2a–d). In sham animals, both male and female mice showed comparable levels of cortical and DG immunoreactivity, with no significant differences between VH and FMT groups (Figures 2e,f). Quantitative analysis of 6E10 immunoreactivity revealed a significant increase in cortical plaque load in both TBI males (####*p* < 0.0001 for VH and FMT groups) and females (###*p* < 0.001 for FMT group) compared to their sham counterparts, with a greater increase in TBI-VH males compared to females (*p < 0.05) (Figure 2e). Moreover, Figure 2e also shows that FMT reduced the 6E10 immunoreactivity in VH-treated TBI-male mice (^★^*p* < 0.05). In the DG, both male and female TBI groups significantly displayed elevated 6E10 + area relative to sham controls (Figure 2f). FMT did not significantly alter amyloid levels in sham or TBI groups. The expression of 6E10 shows in the form (Figures 2a1–d4) of Aβ deposits with plaque morphology. To further characterize plaque maturation, Aβ deposits were classified into three morphologies (Figure 2g): diffuse plaques (large, ill-defined), compact plaques (sharply delineated), and dense-core plaques (fibrillar, with a compact core and surrounding halo) (Thal et al., 2002; Serrano-Pozo et al., 2011; Perez-Nievas et al., 2013; Viejo et al., 2022). Dense-core plaques have been associated with neuritic dystrophy and gliosis, reflecting advanced AD pathology (Serrano-Pozo et al., 2011). In the cortex, TBI induced a marked decrease in total plaque numbers in male mice compared to Sham controls (#*p* < 0.05 for VH and FMT groups) (Figure 2h). Subtype analysis further revealed that dense-core plaques were significantly reduced in Sham males treated with FMT compared to VH (^★^*p* < 0.05) (Figure 2h). Similarly, Sham females receiving FMT showed decreases in diffuse and dense-core Aβ burden relative to VH controls (^★^*p* < 0.05, respectively), whereas no treatment differences were observed in the TBI groups (Figure 2j). In the DG, morphological analysis indicated that in females, significant differences were observed in the diffuse subtype, with a reduction in Sham FMT compared to Sham VH mice (^★^*p* < 0.05) (Figure 2k) that was absent in TBI groups and in males (Figure 2i).

Taken together, these findings demonstrate that injured brains in 5xFAD mice exhibit increased amyloid pathology in the cortex and DG in a sex-dependent manner, with males showing greater susceptibility in the cortex. Importantly, the effects of FMT were most apparent in Sham AD mice, where amyloid deposition was reduced. In contrast, in TBI animals, the neuroinflammatory and neurodegenerative environment appeared to override potential microbiome-mediated benefits.

### 3.3 FMT reduces glial activation in the cortex of 5xFAD male mice following TBI

**Figure 3:**
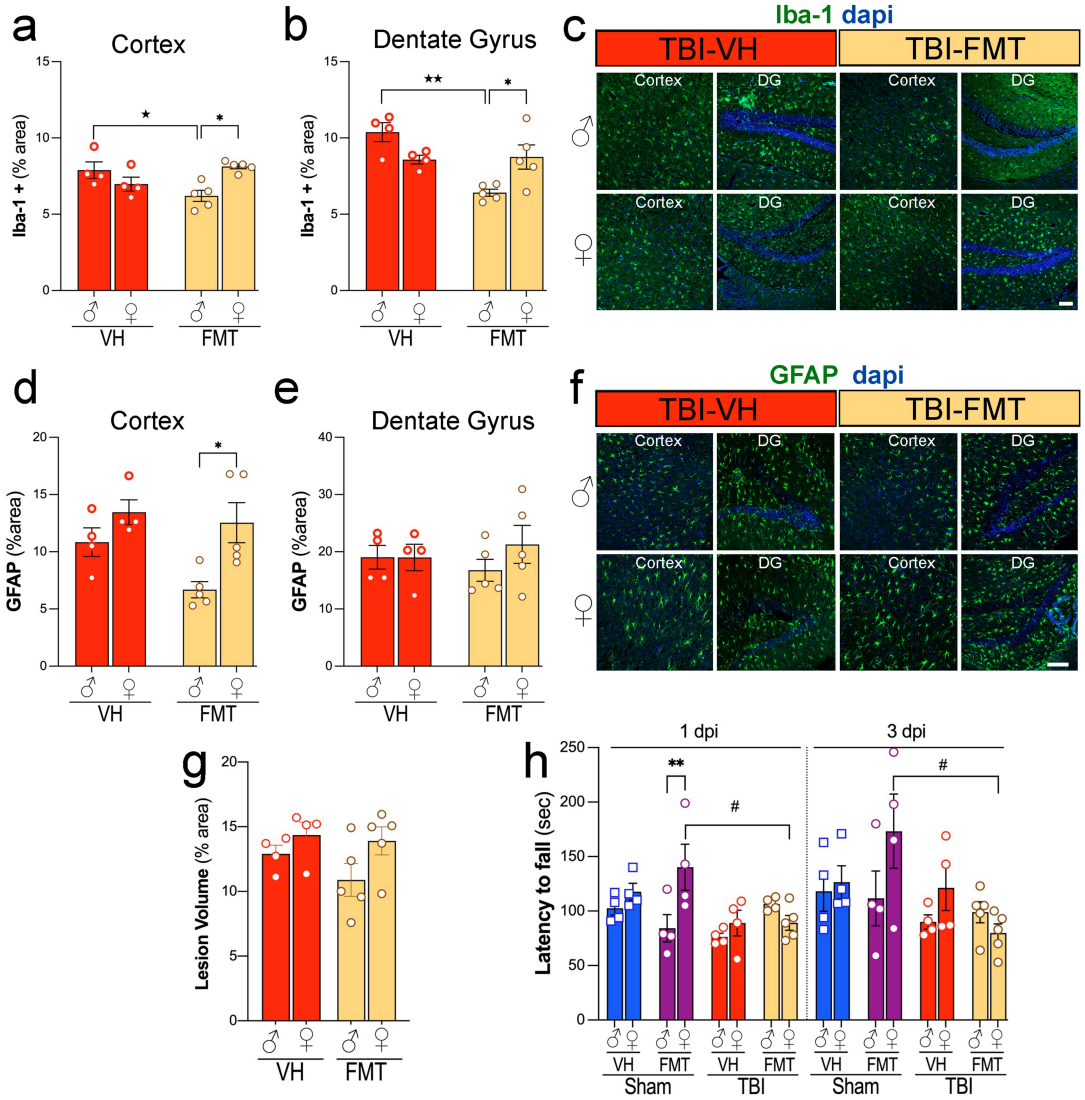
Sex-dependent effects of FMT on microgliosis, astrogliosis, lesion volume, and motor performance after TBI. **(a,b)** Quantification of Iba1+ microglial immunoreactivity (% area) in cortex **(a)** and dentate gyrus **(b)** of male and female TBI-VH and TBI-FMT mice at 3 dpi. FMT reduced Iba1+ area in males in both regions. **(c)** Representative Iba-1 (green) and DAPI (blue) staining in cortex and dentate gyrus from male and female TBI-VH and TBI-FMT mice. **(d,e)** GFAP+ astrocytic immunoreactivity (% area) in cortex **(d)** and dentate gyrus **(e)** shows a modest reduction in cortical GFAP in female TBI-FMT mice compared with TBI-VH, with no major treatment effects in DG. **(f)** Representative GFAP (green) and DAPI (blue) staining. **(g)** Lesion volume (% area) did not differ significantly between VH and FMT groups in either sex. **(h)** Rotarod performance (latency to fall) at 1 and 3 dpi in Sham-VH, Sham-FMT, TBI-VH, and TBI-FMT mice, showing sex- and treatment-dependent motor impairment, with reduced latency in FMT-treated females after TBI. Data are mean ± SEM. Statistics: two-way ANOVA (Iba-1, GFAP, lesion volume) and three-way ANOVA (rotarod) with Bonferroni correction. Symbols indicate significant effects: *sex differences, #Sham vs. TBI, ★treatment (VH vs. FMT). Scale bars: 50 μm.

To evaluate the effect of FMT on neuroinflammation after injury, we quantified Iba-1 and GFAP immunoreactivity in the cortex and DG of 5xFAD mice. Following TBI, male mice treated with FMT showed decreased Iba-1+ microglial activation in the cortex and DG compared to VH controls. However, FMT treatment had no effect in female mice (Figures 3a,b,c). GFAP+ astrocytic reactivity was decreased in male TBI mice treated with FMT compared to females in the cortex, while no changes were observed in any groups for the DG (Figures 3,d,e,f). These findings indicate that FMT modulates microglial and astrocytic responses after TBI in a sex- and region-specific manner.

Next, we assessed whether FMT influenced structural injury and functional outcomes. Lesion volume, measured as percent damaged cortical area, did not differ significantly between VH- and FMT-treated TBI groups in either sex (Figure 3g). However, motor performance on the rotarod revealed that Sham females treated with FMT exhibited improved latency to fall compared to VH at 1 dpi whereas in TBI groups, FMT treatment was associated with reduced performance at both 1 and 3 dpi compared to FMT-treated Sham mice (#*p* < 0.05) (Figure 3h). Moreover, Sham-FMT female mice performed significantly better in the rotarod test compared to Sham-FMT male mice at 1 dpi (**p < 0.01).

### 3.4 FMT modifies gut microbial community profiles after TBI without altering alpha diversity or richness

**Figure 4:**
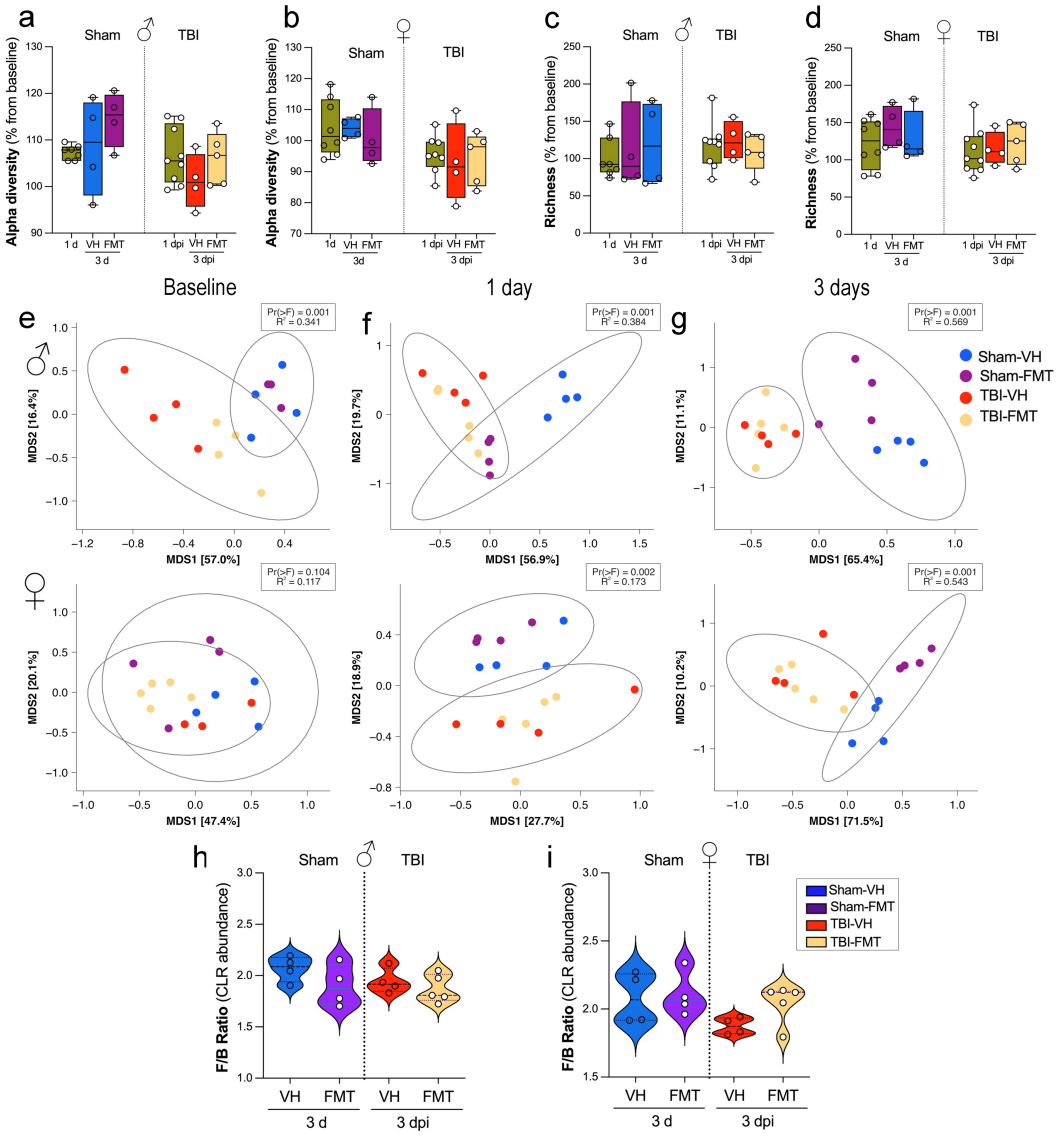
Effects of TBI and FMT on gut microbiota diversity and *Firmicutes/Bacteroidota* ratio. **(a,b)** Alpha diversity (Shannon index, expressed as % change from each animal’s baseline) in male **(a)** and female **(b)** mice at 1 and 3 days after Sham or TBI surgery with VH or FMT treatment. **(c,d)** Richness (observed taxa, % change from baseline) in male **(c)** and female **(d)** mice at the same time points. Boxplots show median and interquartile range; individual points represent mice. Alpha diversity and richness were analyzed by Kruskal–Wallis tests; no significant group differences were detected. **(e–g)** Beta diversity of gut communities visualized by multidimensional scaling (MDS) of Bray–Curtis distances at baseline **(e)**, 1 dpi **(f)**, and 3 dpi **(g)** for males (top) and females (bottom). Ellipses indicate 95% confidence intervals for group centroids. PERMANOVA *p* values and R^2^ are shown for comparisons across the four treatment groups. **(h,i)** *Firmicutes/Bacteroidota* (F/B) ratio (CLR-transformed abundance) at 3 dpi in male **(h)** and female **(i)** mice. Violin plots show the distribution of values; white circles indicate individual animals and dashed lines indicate group means.

To investigate the impact of TBI and FMT on gut microbiota, we first assessed alpha diversity and richness across experimental groups. We investigated the effects of FMT on gut microbial diversity and composition in 5xFAD mice subjected to sham or TBI procedures. Measures of alpha diversity (Shannon index) revealed no significant differences between groups in either males (Figure 4a) or females (Figure 4b) at 1 or 3 dpi. Similarly, microbial richness was not significantly altered across experimental groups at either time point (Figures 4c,d), with or without FMT treatment. We next evaluated beta diversity using principal coordinate analysis (PCoA) based on Bray–Curtis dissimilarity (Figures 4e–g), showing marked group-dependent effects. At baseline, microbial communities did not differ significantly among groups (Figure 4e; Pr(>F) = 0.001, R2 = 0.341). However, by 1 dpi, significant separation was observed across sham and TBI groups (Figure 4f; Pr(>F) = 0.001, R^2^ = 0.384), with clustering driven by both injury and FMT status. This effect was more pronounced at 3 dpi (Figure 4g; Pr(>F) = 0.001, R^2^ = 0.569), where distinct microbiome profiles were evident between sham and TBI mice, and between VH and FMT-treated animals, indicating that donor microbiota exerted a measurable effect on post-injury microbial engraftment.

Together, these results indicate that while FMT and TBI do not significantly alter alpha diversity or richness, they strongly reshape the overall microbial community structure, with effects becoming increasingly evident over time after injury. FMT partially modulates these shifts, suggesting that microbiota restoration strategies may counteract aspects of TBI-induced dysbiosis in a sex-dependent manner.

### 3.5 FMT modulates microbial taxonomic composition dynamics following TBI

**Figure 5:**
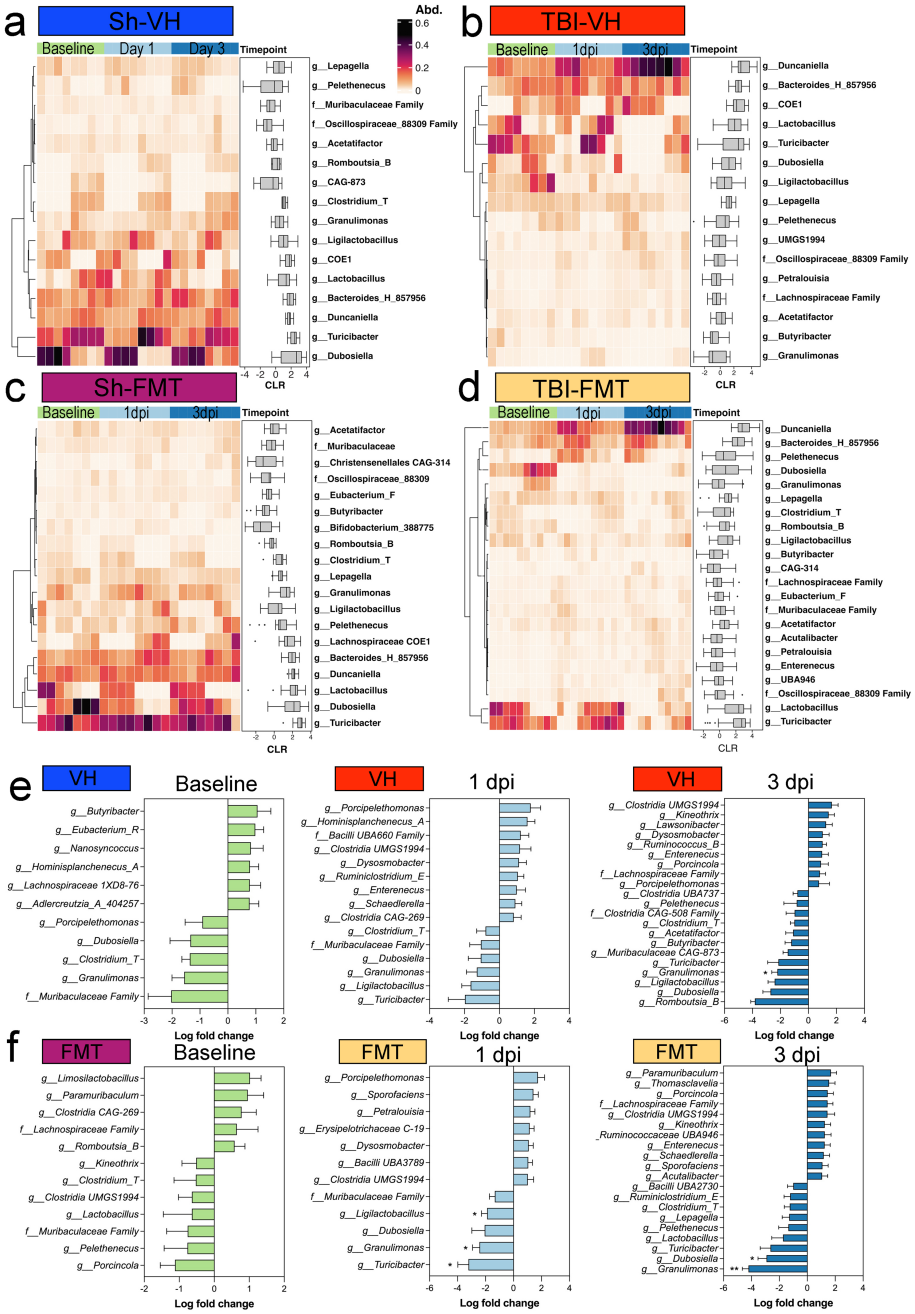
Taxonomic shifts in fecal microbiota after TBI and FMT. Heatmaps showing relative abundance (Abd.) of selected bacterial genera at baseline, 1 dpi, and 3 dpi in **(a)** Sham-VH, **(b)** TBI-VH, **(c)** Sham-FMT, and **(d)** TBI-FMT groups. Color scale indicates relative abundance (0–0.6). Boxplots adjacent to each heatmap display centered log-ratio (CLR)-transformed abundances over time for key taxa. **(e,f)** Differential abundance analysis comparing sham and TBI mice within VH **(e)** or FMT **(f)** treatment at each time point, shown as log fold change (TBI vs. sham). Negative values indicate decreased abundance in TBI, positive values indicate enrichment. In TBI-FMT mice, reductions were observed in *Turicibacter, Granulimonas,* and *Ligilactobacillus* at the 1 dpi timepoint, with the altered *Granulimonas* persisting into the 3 dpi timepoint of both VH and FMT groups.

To investigate specific microbial shifts associated with FMT after TBI, we analyzed genus-level relative abundances across groups and time points. Heatmap clustering revealed distinct temporal patterns in Sham VH, TBI VH, Sham FMT, and TBI FMT mice (Figures 5a–d). Sham VH mice showed relatively stable microbial profiles over time, with moderate fluctuations in genera such as *Lepageella*, *Pelethenecus*, *Muribaculaceae* family members, *Oscillospiraceae*, and *Acetatifactor*, were consistently detected across timepoints (Figures 5a,e). In contrast, TBI VH mice exhibited marked temporal changes, with increased abundance of *Duncaniella*, *Bacteroides_H_857956*, and *Ligilactobacillus* by 3 dpi (Figure 5b) and with a decrease in beneficial taxa such as *Lactobacillus*, *Turicibacter*, and *Dubosiella* (Figures 5b,d). FMT treatment reshaped microbial trajectories in both sham and injured animals. Sham FMT mice demonstrated enrichment of beneficial taxa, including *Bifidobacterium*, *Butyricicoccus*, and *Christensenellaceae* family members, particularly by 3 dpi (Figure 5c). In TBI FMT mice, the microbial community was dominated by reduction in SCFA-producing taxa, including *Turicibacter*, *Granulimonas*, and *Ligilactobacillus*, when comparing Sham and TBI cohorts (Figures 5e,f). FMT treatment produced distinct patterns of microbial remodeling depending on the condition. At 1 dpi, VH mice exhibited only minor, non-significant changes in bacterial genera. In contrast, FMT-treated mice showed a significant reduction in SCFA-producing taxa, including *Turicibacter*, *Granulimonas*, and *Ligilactobacillus*, when comparing Sham and TBI cohorts (Figures 5e,f). By 3 dpi, differences between Sham and TBI groups persisted: *Granulimonas* remained reduced, and another key gut–brain axis taxon, *Dubosiella*, was significantly depleted in FMT-treated TBI animals. Together, these findings indicate that TBI disrupts gut microbial composition by selectively enriching pro-inflammatory species while depleting protective commensals. FMT partially counteracts these effects by supporting restoration of beneficial taxa, particularly SCFA producers such as *Dubosiella*. These taxonomic shifts suggest potential mechanisms by which microbiota modulation may influence host neuroinflammatory outcomes following TBI.

### 3.6 TBI or FMT did not significantly alter SCFA levels

**Figure 6:**
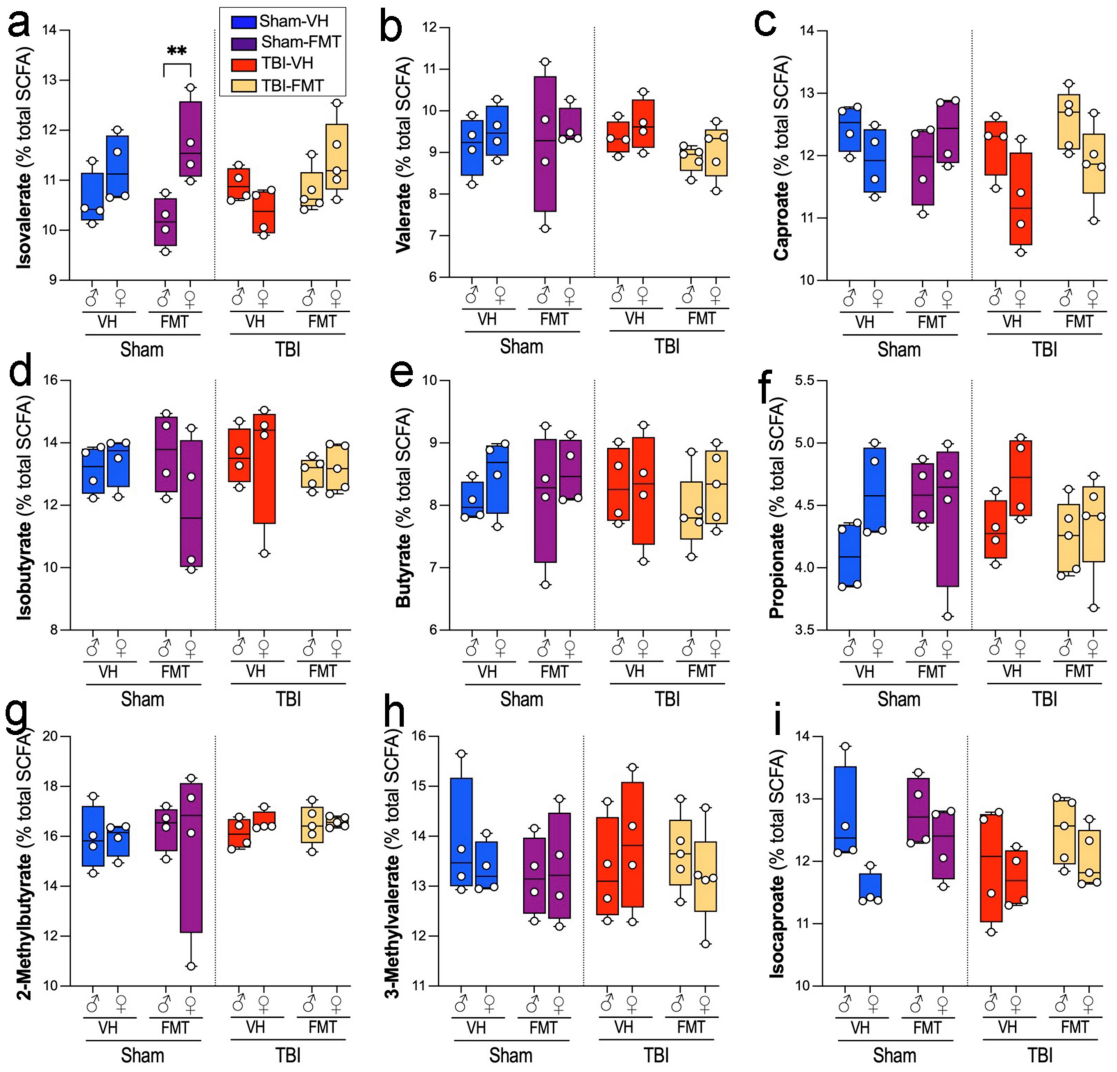
Effects of TBI and FMT on fecal short-chain fatty acid (SCFA) profiles. Relative abundance of individual serum SCFAs (percentage of total SCFA pool) in male and female 5xFAD mice subjected to Sham or TBI surgery and treated with VH or FMT. Panels show: **(a)** Isovalerate, **(b)** Valerate, **(c)** Caproate, **(d)** Isobutyrate, **(e)** Butyrate, **(f)** Propionate, **(g)** 2-methylbutyrate, **(h)** 3-methylvalerate, and **(i)** Isocaproate. Boxplots show median and interquartile range with individual data points. Sham-FMT females exhibited higher isovalerate levels than Sham-FMT males (**p < 0.01), whereas no significant differences were observed between TBI groups or for other SCFAs. Data are mean ± SEM overlaid on boxplots for visualization. Statistics: three-way ANOVA with Bonferroni correction. Symbols indicate significant effects: *sex differences, #Sham vs. TBI.

We next examined how FMT affected serum SCFA composition in sham and TBI 5xFAD mice. Among the measured SCFAs, isovalerate levels were significantly elevated in Sham-FMT in females compared to male mice (Figure 6a; **p < 0.01). Other SCFAs, including valerate (Figure 6b), caproate (Figure 6c), isobutyrate (Figure 6d), butyrate (Figure 6e), propionate (Figure 6f), 2-methylbutyrate (Figure 6g), 3-methylvalerate (Figure 6h), and isocaproate (Figure 6i) remained unchanged across treatments, sexes and injury conditions.

### 3.7 FMT modulates gut morphology in 5xFAD male mice

**Figure 7:**
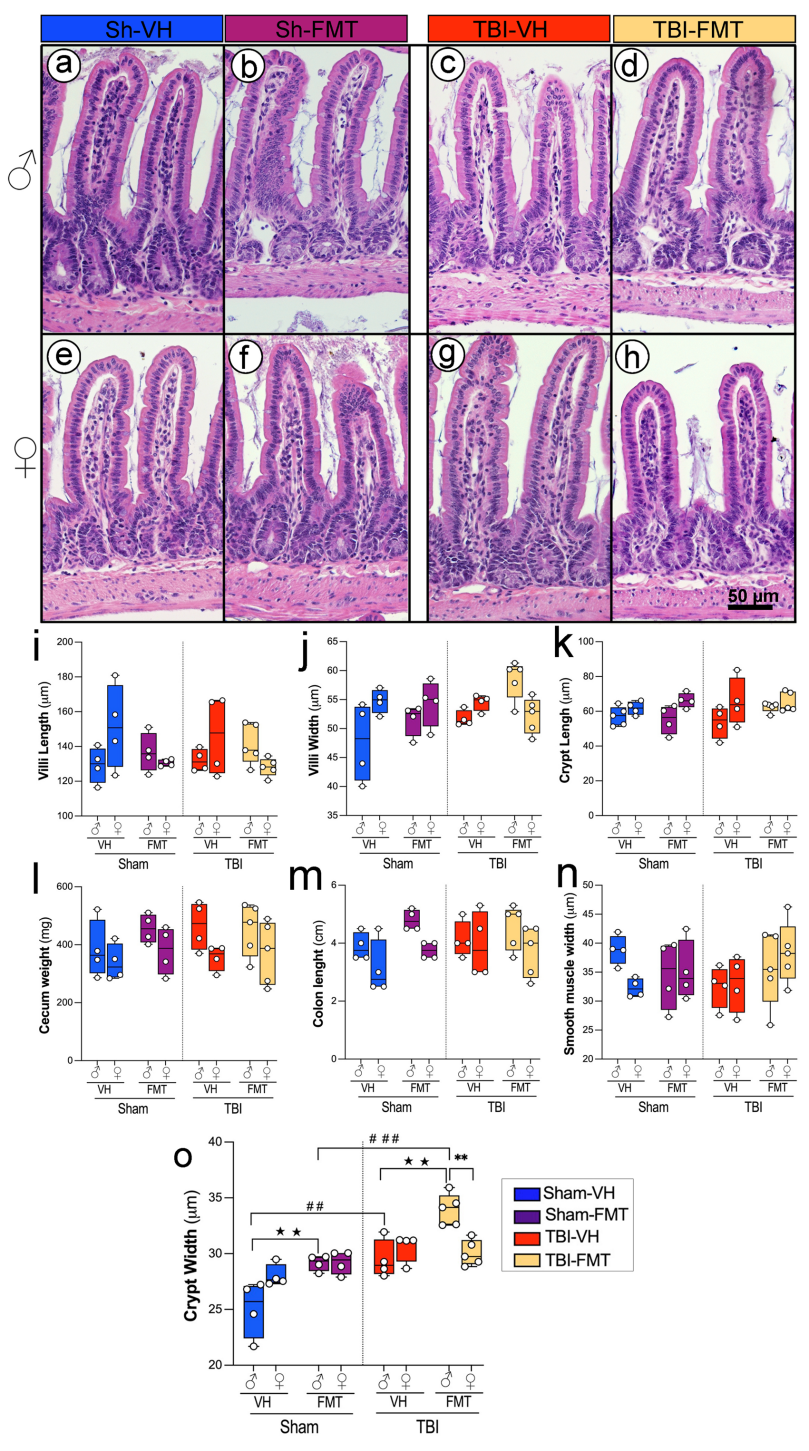
Effects of TBI and FMT on intestinal morphology in male and female 5xFAD mice. Representative H&E-stained sections of small intestinal villi and crypts from male **(a–d)** and female **(e–h)** mice in Sham-VH **(a,e)**, Sham-FMT **(b,f)**, TBI-VH **(c,g)**, and TBI-FMT **(d,h)** groups. Quantification of **(i)** villus length, **(j)** villus width, and **(k)** crypt length shows no major differences between groups. **(l–n)** Gross gut anatomy, including cecum weight **(l)**, colon length **(m)**, and smooth muscle thickness **(n)**, was not significantly altered by TBI or FMT. **(o)** Crypt width was significantly increased in TBI mice compared with Sham controls, particularly in VH- and FMT-treated males, indicating enhanced epithelial remodeling after injury (★*p* < 0.05, ★★*p* < 0.01; ##*p* < 0.01, ###*p* < 0.001). Data are shown as box-and-whisker plots with individual values. Symbols indicate significant effects: *sex differences, #Sham vs. TBI, ★treatment (VH vs. FMT). Scale bar: 50 μm.

To investigate whether TBI and FMT influence gut structural integrity, we analyzed histological parameters of the small intestine and colon using H&E-stained sections (Figures 7a–h). Representative H&E images revealed preserved villus and crypt structures across all groups, although subtle alterations were evident between sham and TBI conditions.

Quantitative analysis showed no significant differences in villus length or villus width across groups (Figures 7i,j). Similarly, crypt length remained unchanged in both sham and TBI animals regardless of treatment (Figure 7k). Other gross measures of gut anatomy, including cecum weight, colon length, and smooth muscle thickness, did not differ significantly between experimental groups (Figures 7l−n). In contrast, crypt width was significantly increased in TBI mice compared to sham controls, particularly in males with both VH and FMT treatments (Figure 7l,o; ##*p* < 0.01, ###*p* < 0.001). FMT treatment increased crypt width only in males in both Sham and TBI groups (★★*p* < 0.01). In addition, in the TBI group, an increase is observed in males compared to females only in FMT-treated mice, suggesting that FMT modifies epithelial architecture in a sex-dependent manner (**p < 0.01).

Together, these results demonstrate that while overall gut architecture remains intact following TBI, crypt morphology is selectively altered, and FMT modulates these changes, suggesting a sex-specific susceptibility and protective role in maintaining epithelial structure after injury.

## 4 Discussion

In this study, we subjected AD mouse models to TBI and found that the injury accelerated amyloid pathology, particularly in females. A short-term, single FMT administration was not sufficient to counteract the TBI-induced neuroinflammatory response, although notable effects on the amyloid pathology were observed in sham AD mice. These findings indicate that a single FMT intervention cannot fully reverse TBI-associated neuroinflammation, but it does elicit sex-dependent alterations in brain amyloid pathology and microbiome profiles.

### 4.1 TBI exacerbates amyloid pathology and neuroinflammation in a sex-dependent manner

Consistent with previous reports linking TBI to accelerated AD progression (Ramos-Cejudo et al., 2018), our results show increased cortical amyloid accumulation, increased Aβ plaques, and impaired motor performance. These effects were more pronounced in females, reinforcing clinical and preclinical evidence of heightened vulnerability to AD pathology in females (Zhu et al., 2021; Vila-Castelar et al., 2025). Our findings demonstrate that TBI significantly increased overall Aβ6E10 immunoreactivity in the cortex and hippocampus of both male and female mice. Notably, this increase in total amyloid burden was accompanied by a relative reduction in compact and dense-core plaques. Given that soluble Aβ oligomers are widely regarded as the most neurotoxic species, whereas dense-core fibrillar plaques may serve to sequester soluble forms, this morphological shift suggests altered aggregation dynamics rather than a protective effect (Goure et al., 2014; Xu et al., 2020). Rather than mitigating pathology, the reduction of dense-core plaques after TBI may indicate impaired sequestration of soluble Aβ, thereby favoring accumulation of oligomeric and diffusely aggregated forms that are more harmful to AD pathology. Thus, TBI appears to exacerbate amyloid pathology through enhanced accumulation of aggregated Aβ, implicating secondary injury mechanisms in the worsening of neurodegenerative processes.

At the molecular level, these changes are consistent with TBI-induced activation of inflammatory and oxidative pathways (e.g., NF-κB and NLRP3 inflammasome signaling, mitochondrial dysfunction, and BBB breakdown) that can upregulate β- and *γ*-secretase activity and dysregulate tau kinases such as GSK3β and CDK5, collectively promoting Aβ overproduction, impaired clearance, and tau hyperphosphorylation. In the 5xFAD model, all animals received a standardized, moderate CCI, and our design was not powered to detect a graded correlation between individual injury severity and plaque burden. However, studies in Tg2576 mice have shown that repetitive mild TBI, but not a single mild TBI, increases amyloid deposition (Uryu et al., 2002), and epidemiological data in humans support a severity-dependent relationship between head injury and subsequent AD risk (Guo et al., 2000). Our data therefore fit within a framework in which TBI severity and repetition modulate the extent of AD-like pathology, while our study specifically captures the consequences of a single, standardized injury.

Females consistently develop more severe pathology in AD mouse models, including the 5xFAD mice (Sil et al., 2022). Although the underlying mechanisms are not yet fully elucidated, sex-specific differences in hormonal regulation (e.g., estrogen/progesterone effects on microglia, APP processing, and synaptic function), inflammatory signaling, and gene expression are thought to contribute to this dimorphism (Carroll et al., 2010). Baseline sex differences in microbiota composition and immune-microbiome crosstalk may further shape the response to TBI. Together with our previously reported sex differences in immune responses after TBI (Villapol et al., 2017), these observations highlight the need to develop sex-specific, microbiome-targeted therapeutic strategies.

### 4.2 Impact of FMT on the neuropathology post-TBI

While prior studies reported that FMT-treated mice exhibited significant preservation of cortical volume and white matter connectivity at chronic time points, such as 60 dpi (Davis et al., 2022a) or 90 dpi (Du et al., 2021), our findings did not reveal comparable effects during the acute phase. However, the interpretation of the mouse study warrants cautions due to limited statistical power. In a comparable pig model, FMT modestly enhanced recovery rates as reflected by gross MRI pathology markers and motor function (Fagan et al., 2023). Beyond their therapeutic potential, FMT has also been employed as an investigative tool to study disease mechanisms and pathology rather than solely as an intervention for recovery.

In this study, the absence of any effect on lesion size reinforces the idea that the benefits of FMT arise not from direct neuroprotection against acute mechanical trauma, but rather from modulation of secondary injury cascades. Consistent with this, our findings indicate that microbiome modulation may influence the qualitative features of amyloid pathology, such as plaque morphology, rather than entirely preventing the trajectory of Aβ deposition. This effect warrants further mechanistic investigation. TBI induced robust microgliosis and astrogliosis in both the cortex and dentate gyrus. Yet, FMT exerted significant effects, selectively attenuating microglial activity in the cortex and DG in male mice while leaving astrocytic reactivity largely unchanged. Despite these histological changes, FMT did not reduce lesion volume or improve motor outcomes, suggesting that microbiota-based interventions alone may be insufficient to alter gross injury outcomes in this aggressive AD model. Although females also exhibited greater TBI-induced amyloid pathology, they showed less microglial suppression and distinct microglial remodeling in response to FMT compared with males. These sex-specific differences likely arise from intersecting effects of sex hormones on microglial activation, sex-biased immune signaling pathways, and underlying differences in microbiota composition and function (Markle et al., 2013; Jasarevic et al., 2016; Klein and Flanagan, 2016; Org et al., 2016; Villa et al., 2018). Collectively, our data support the notion that FMT and related microbiome-based interventions may have sex-dependent efficacy in restoring AD-related pathology after TBI, reinforcing the need for sex-stratified design and analysis in preclinical and clinical microbiota-targeted therapies.

### 4.3 TBI disrupts microbiota beta diversity

TBI has been shown to reduce microbial diversity and richness, consistent with post-injury dysbiosis observed in both human and rodent studies (Treangen et al., 2018; Nicholson et al., 2019). In our AD model, however, we did not detect such differences following TBI, reflecting variability among samples and the limited number of mice analyzed. Analysis of microbial communities revealed that TBI altered beta diversity in a time-dependent manner. Differences emerged as early as day 1 and became more pronounced by day 3, with apparent clustering of TBI and sham communities. These shifts were also sex-dependent, with females showing more pronounced reductions in richness following TBI. Community separation, evident at 1- and 3-dpi, suggests rapid and sustained ecological disruption driven by systemic inflammation, altered gut motility, and stress-related hormonal changes (El Baassiri et al., 2024).

Because samples were collected at a single acute post-injury window, we cannot yet determine whether these compositional shifts are transient adaptations or stable reconfigurations of the microbiome. Longitudinal sampling will be required to assess the durability and potential reversibility of these changes. Nonetheless, the observed beta-diversity alterations are consistent with functional consequences: changes in taxa with known roles in SCFA production, barrier maintenance, and immune modulation are poised to affect metabolic outputs and peripheral and central immune tone. While our current dataset provides only partial evidence for such links (e.g., modest SCFA correlations), more comprehensive metabolomic and immunophenotyping approaches will be needed to determine how these community-level changes translate into measurable metabolic or immune effects in the brain and periphery.

### 4.4 16S rRNA profiling identified distinct taxonomic signatures associated with TBI and FMT, with no differences in SCFA production

FMT from healthy donors reshaped the gut microbial community, but its effects were highly context dependent. Several studies demonstrate that FMT can ameliorate TBI-induced neuropathology, attenuating lesion size, ventriculomegaly, and white matter loss while reducing microglial inflammatory gene expression (Du et al., 2021; Davis et al., 2022a, 2022b). Translational studies extend these findings to large animal models, where FMT limited neuronal loss and improved motor outcomes in pediatric piglets (Fagan et al., 2023), highlighting its clinical potential. This sex-specific divergence highlights underlying differences in microbiome stability and responsiveness, which may contribute to distinct neuroinflammatory and pathological outcomes between males and females. In this study, there were no significantly altered taxa between the sham and TBI groups in either the VH or FMT animals at baseline. By 1 dpi, however, the TBI FMT group showed a significant decrease in *Ligilactobacillus*, *Granulimonas*, and *Turicibacter,* whereas no such significant taxonomic alterations were seen in the sham-TBI comparison of the VH group. At 3 dpi, both VH and FMT TBI mice presented with significantly decreased *Granulimonas*, with the FMT TBI mice showing significantly decreased *Dubosiella*. The reduction in both *Granulimonas* and *Dubosiella* suggests that the altered metabolic environment of the injured gut may create distinct ecological niches that favor different donor-derived microbes (Everard et al., 2013; O'Callaghan and van Sinderen, 2016). These divergent outcomes underscore that the host context critically shapes colonization dynamics, helping to explain why FMT can be beneficial in some settings yet neutral or even harmful in others. Notably, cross-sex FMT (male donors into female recipients after TBI, and vice versa) has been shown to produce distinct, sex-specific shifts in the microbiome (Pasam and Dandekar, 2023), further supporting the concept that sex moderates microbiota-based interventions. To complement taxonomic analyses, we also quantified high-level phylum composition and calculated the Firmicutes/Bacteroidota (F/B) ratio. Although the F/B ratio is a coarse metric, often linked to metabolic status and inflammatory tone, however we did not observe any differences.

Functional analyses of microbial metabolites provided additional insights. SCFAs, including acetate, propionate, and butyrate, are critical regulators of epithelial barrier integrity, microglial maturation, and neuroinflammatory tone (Erny et al., 2015). SCFA levels, particularly butyrate and isobutyrate, correlated with improved motor function and reduced neuroinflammation, supporting their role as neuroprotective metabolites (Dalile et al., 2019). However, we did not observed changes in the SCFA levels to sham values or treatment. These patterns can be disrupted by TBI-induced anorexia, impaired gut motility, and accelerated mucosal turnover (Opeyemi et al., 2021). Female sham-FMT animals only showed higher isovalerate compared with males, suggesting sex-specific metabolic outputs. These patterns suggest a potential functional consequence of microbiota shifts that may require larger cohorts or more extended observation periods to confirm. Beyond SCFAs, other microbiota-derived metabolites, such as trimethylamine N-oxide, secondary bile acids, and tryptophan-derived molecules (including serotonin, kynurenine, and indoles) have also been shown to influence amyloid processing and plaque maturation (Kang et al., 2024). Beyond SCFAs, other microbiota-derived metabolites, such as trimethylamine N-oxide, secondary bile acids, and tryptophan-derived molecules (including serotonin, kynurenine, and indoles), have been shown to influence microglial activation, amyloid processing, and plaque maturation (Kang et al., 2024). Our study did not directly quantify these metabolites, but their known roles highlight plausible routes through which FMT-induced taxonomic changes could affect plaque morphology and neuroinflammation. Finally, while certain taxa and metabolite profiles in our dataset are associated with more “neuroprotective” versus “neurotoxic” outcomes, our 16S and SCFA data are correlative and do not allow us to identify causal taxa or metabolites. Definitive attribution of neuroprotective or neurotoxic effects to specific microbes or metabolites will require gnotobiotic transfers, defined consortia, and targeted supplementation or inhibition of specific metabolites.

### 4.5 TBI and FMT reshape intestinal morphology

TBI-induced neuropathological changes occurred alongside intestinal structural alterations, villus atrophy, crypt hypertrophy, and increased crypt width, supporting prior findings that brain injury rapidly disrupts gut homeostasis (Opeyemi et al., 2021). Our results demonstrate that TBI increases crypt width in males, but not in females, an indicator often associated with epithelial hyperplasia in response to injury or inflammation (Hang et al., 2003).

Rapid crypt proliferation and turnover traverse up the villi to replace damaged epithelia; loss or exhaustion of this proliferative response has been noted in inflammatory diseases (Gersemann et al., 2011; Clevers, 2013; Barker, 2014).

FMT seems to enhance this regenerative process, morphologically seen through crypt widening. This increase was evident in the sham male mice and was further exacerbated by TBI. FMT amplified some of these changes, particularly increasing villus width in TBI mice. Mechanistically, FMT-induced crypt widening could reflect adaptive epithelial remodeling driven by altered microbial metabolites and growth factors, or alternatively a pro-inflammatory mucosal state characterized by increased immune cell infiltration and epithelial stress (Luissint et al., 2016).

These findings suggest that microbiota transfer affects epithelial remodeling processes, potentially through altered microbial signaling or metabolite production. TBI may further weaken morphological function for proper immune integration, which is potentially restored or reshaped by FMT and results in drastic epithelial adjustments. The significant increase in crypt width after individual FMT and combined FMT-TBI exposure suggests that FMT treatment may induce short-term intestinal inflammation and epithelial remodeling, perhaps through microbial competition to re-establish a heathy gut microbiome (Pu et al., 2024). Interestingly, colon length, smooth muscle thickness, and cecum weight remained unchanged, indicating that changes were localized to epithelial than global gut anatomy. Restoration of gut structure, particularly normalization of villus and crypt architecture and reinforcement of epithelial integrity, may reduce systemic inflammation and microbial translocation, thereby dampening the “leaky gut,” neuroinflammation cascade. However, the current study does not address whether these morphological changes are reversible over longer recovery periods. Future work should incorporate longitudinal histology and functional assays of barrier integrity (e.g., tight junction markers, permeability tests) to determine whether FMT-induced crypt widening represents a transient adaptive response or a persistent maladaptive phenotype.

### 4.6 Implications and future directions

A key limitation of this study is the short post-treatment period after TBI, which captures acute but not chronic changes in the microbiome, metabolites, and neuropathology. Longitudinal studies will be essential to determine whether the effects of early FMT persist, resolve, or evolve over time, and whether beta-diversity shifts represent transient perturbations or stable ecological transitions. Expanded metabolomic profiling, including bile acids, amino acid derivatives, neurotransmitter precursors, and other microbiota-derived metabolites implicated in plaque maturation, could provide a more complete functional landscape of gut microbial activity post-TBI.

Another limitation is the relatively small sample size. Pooling mice by sex, while necessary for statistical power, may reduce sensitivity to detect subtler but biologically meaningful differences within highly interconnected systems such as the gut microbiome. Moreover, our 16S and SCFA data do not permit causal inference; they instead highlight candidate taxa and metabolite classes that can be prioritized for mechanistic follow-up.

Mechanistic studies using germ-free mice or microbiota depletion before FMT treatment could help clarify causal pathways linking microbiota modulation to amyloid pathology and neuroinflammation. Given that FMT outcomes are donor-dependent and sex-dependent, future work should aim to identify specific microbial strains or metabolites responsible for neuroprotection, enabling more targeted and standardized therapies and explicitly testing sex-specific efficacy. At the microbial species level, transplantation of *Prevotella copri* was shown to restore gut homeostasis and protect against oxidative stress (Gu et al., 2024), further supporting the therapeutic capacity of targeted single microbial interventions. Complementary metabolomic approaches identified natural compounds derived from gut microbial metabolism as potent inhibitors of TBI-induced microglial NLRP3 inflammasome activation, which should be interesting to apply in this context (Tang et al., 2025).

## 5 Conclusion

Collectively, our findings demonstrate that TBI accelerates amyloid pathology, neuroinflammation, and gut dysbiosis in 5xFAD mice, with females exhibiting greater vulnerability. Although FMT partially enriched putatively beneficial taxa, modified plaque morphology, and altered intestinal architecture, it did not prevent amyloid accumulation, microgliosis, or behavioral impairments in this aggressive AD model during the acute phase. These results reinforce the gut-brain axis as a critical mechanistic link between TBI and AD and point to the need for longer-term, combinatorial, and sex-tailored microbiome-based interventions to achieve sustained neuroprotection.

## Data Availability

The data presented in this study are publicly available. This data can be found here: https://www.ncbi.nlm.nih.gov/sra, accession PRJNA1220689. All code used within the analysis of the microbiome 16S rRNA sequencing data can be found at https://github.com/villapollab/fmt_ad_no_abx.

## References

[ref1] Barker N. (2014). Adult intestinal stem cells: critical drivers of epithelial homeostasis and regeneration. Nat. Rev. Mol. Cell Biol. 15, 19–33. doi: 10.1038/nrm3721, 24326621

[ref2] Caporaso J. G. Lauber C. L. Walters W. A. Berg-Lyons D. Huntley J. Fierer N. . (2012). Ultra-high-throughput microbial community analysis on the Illumina HiSeq and MiSeq platforms. ISME J. 6, 1621–1624. doi: 10.1038/ismej.2012.8, 22402401 PMC3400413

[ref3] Carroll J. C. Rosario E. R. Kreimer S. Villamagna A. Gentzschein E. Stanczyk F. Z. . (2010). Sex differences in β-amyloid accumulation in 3xTg-AD mice: role of neonatal sex steroid hormone exposure. Brain Res. 1366, 233–245. doi: 10.1016/j.brainres.2010.10.009, 20934413 PMC2993873

[ref4] Celorrio M. Abellanas M. A. Rhodes J. Goodwin V. Moritz J. Vadivelu S. . (2021). Gut microbial dysbiosis after traumatic brain injury modulates the immune response and impairs neurogenesis. Acta Neuropathol. Commun. 9:40. doi: 10.1186/s40478-021-01137-2, 33691793 PMC7944629

[ref5] Clevers H. (2013). The intestinal crypt, a prototype stem cell compartment. Cell 154, 274–284. doi: 10.1016/j.cell.2013.07.004, 23870119

[ref6] Dalile B. Van Oudenhove L. Vervliet B. Verbeke K. (2019). The role of short-chain fatty acids in microbiota-gut-brain communication. Nat. Rev. Gastroenterol. Hepatol. 16, 461–478. doi: 10.1038/s41575-019-0157-3, 31123355

[ref7] Davis B. T. Chen Z. Islam M. Timken M. E. Procissi D. Schwulst S. J. (2022a). Fecal microbiota transfer attenuates gut dysbiosis and functional deficits after traumatic brain injury. Shock 57, 251–259. doi: 10.1097/SHK.0000000000001934, 35759305 PMC10341382

[ref8] Davis B. T. Chen Z. Islam M. Timken M. E. Procissi D. Schwulst S. J. (2022b). Postinjury fecal microbiome transplant decreases lesion size and neuroinflammation in traumatic brain injury. Shock 58, 287–294. doi: 10.1097/SHK.0000000000001979, 36256625 PMC9586470

[ref9] DeTure M. A. Dickson D. W. (2019). The neuropathological diagnosis of Alzheimer's disease. Mol. Neurodegener. 14:32. doi: 10.1186/s13024-019-0333-5, 31375134 PMC6679484

[ref10] Du D. Tang W. Zhou C. Sun X. Wei Z. Zhong J. . (2021). Fecal microbiota transplantation is a promising method to restore gut microbiota dysbiosis and relieve neurological deficits after traumatic brain injury. Oxidative Med. Cell. Longev. 2021:5816837. doi: 10.1155/2021/5816837, 33628361 PMC7894052

[ref11] El Baassiri M. G. Raouf Z. Badin S. Escobosa A. Sodhi C. P. Nasr I. W. (2024). Dysregulated brain-gut axis in the setting of traumatic brain injury: review of mechanisms and anti-inflammatory pharmacotherapies. J. Neuroinflammation 21:124. doi: 10.1186/s12974-024-03118-3, 38730498 PMC11083845

[ref12] Erny D. de Hrabe Angelis A.L. Jaitin D. Wieghofer P. Staszewski O. David E. 2015). Host microbiota constantly control maturation and function of microglia in the CNS. Nat. Neurosci. 18, 965–977. doi: doi: 10.1038/nn.4030, 26030851. 26030851 PMC5528863

[ref13] Everard A. Belzer C. Geurts L. Ouwerkerk J. P. Druart C. Bindels L. B. . (2013). Cross-talk between Akkermansia muciniphila and intestinal epithelium controls diet-induced obesity. Proc. Natl. Acad. Sci. USA 110, 9066–9071. doi: 10.1073/pnas.1219451110, 23671105 PMC3670398

[ref14] Fagan M. M. Welch C. B. Scheulin K. M. Sneed S. E. Jeon J. H. Golan M. E. . (2023). Fecal microbial transplantation limits neural injury severity and functional deficits in a pediatric piglet traumatic brain injury model. Front. Neurosci. 17:1249539. doi: 10.3389/fnins.2023.1249539, 37841685 PMC10568032

[ref15] Flinn H. Marshall A. Holcomb M. Cruz L. Soriano S. Treangen T. J. . (2024). Antibiotic treatment induces microbiome dysbiosis and reduction of neuroinflammation following traumatic brain injury in mice. bioRxiv:2024.2005.2011.593405. doi: 10.1101/2024.05.11.593405

[ref16] Gersemann M. Stange E. F. Wehkamp J. (2011). From intestinal stem cells to inflammatory bowel diseases. World J. Gastroenterol. 17, 3198–3203. doi: 10.3748/wjg.v17.i27.3198, 21912468 PMC3158395

[ref17] Goure W. F. Krafft G. A. Jerecic J. Hefti F. (2014). Targeting the proper amyloid-beta neuronal toxins: a path forward for Alzheimer's disease immunotherapeutics. Alzheimer's Res Ther 6:42. doi: 10.1186/alzrt272, 25045405 PMC4100318

[ref18] Gu N. Yan J. Tang W. Zhang Z. Wang L. Li Z. . (2024). *Prevotella copri* transplantation promotes neurorehabilitation in a mouse model of traumatic brain injury. J. Neuroinflammation 21:147. doi: 10.1186/s12974-024-03116-5, 38835057 PMC11151605

[ref19] Guo Z. Cupples L. A. Kurz A. Auerbach S. H. Volicer L. Chui H. . (2000). Head injury and the risk of AD in the MIRAGE study. Neurology 54, 1316–1323. doi: 10.1212/wnl.54.6.1316, 10746604

[ref20] Hang C. H. Shi J. X. Li J. S. Wu W. Yin H. X. (2003). Alterations of intestinal mucosa structure and barrier function following traumatic brain injury in rats. World J. Gastroenterol. 9, 2776–2781. doi: 10.3748/wjg.v9.i12.2776, 14669332 PMC4612051

[ref21] Harach T. Marungruang N. Duthilleul N. Cheatham V. Mc Coy K. D. Frisoni G. . (2017). Erratum: reduction of Abeta amyloid pathology in APPPS1 transgenic mice in the absence of gut microbiota. Sci. Rep. 7:46856. doi: 10.1038/srep46856, 28691712 PMC5502384

[ref22] Heneka M. T. Carson M. J. El Khoury J. Landreth G. E. Brosseron F. Feinstein D. L. (2015). Neuroinflammation in Alzheimer's disease. Lancet Neurol. 14, 388–405. doi: 10.1016/S1474-4422(15)70016-5, 25792098 PMC5909703

[ref23] Heneka M. T. Kummer M. P. Stutz A. Delekate A. Schwartz S. Vieira-Saecker A. . (2013). NLRP3 is activated in Alzheimer's disease and contributes to pathology in APP/PS1 mice. Nature 493, 674–678. doi: 10.1038/nature11729, 23254930 PMC3812809

[ref24] Heston M. B. Hanslik K. L. Zarbock K. R. Harding S. J. Davenport-Sis N. J. Kerby R. L. . (2023). Gut inflammation associated with age and Alzheimer's disease pathology: a human cohort study. Sci. Rep. 13:18924. doi: 10.1038/s41598-023-45929-z, 37963908 PMC10646035

[ref25] Hickman S. Izzy S. Sen P. Morsett L. El Khoury J. (2018). Microglia in neurodegeneration. Nat. Neurosci. 21, 1359–1369. doi: 10.1038/s41593-018-0242-x, 30258234 PMC6817969

[ref26] Holcomb M. Marshall A. G. Flinn H. Lozano-Cavazos M. Soriano S. Gomez-Pinilla F. . (2025). Probiotic treatment induces sex-dependent neuroprotection and gut microbiome shifts after traumatic brain injury. J. Neuroinflammation 22:114. doi: 10.1186/s12974-025-03419-1, 40254574 PMC12010691

[ref27] Jasarevic E. Morrison K. E. Bale T. L. (2016). Sex differences in the gut microbiome-brain axis across the lifespan. Philos. Trans. R. Soc. Lond. Ser. B Biol. Sci. 371:20150122. doi: 10.1098/rstb.2015.0122, 26833840 PMC4785905

[ref28] Johnson V. E. Stewart W. Smith D. H. (2010). Traumatic brain injury and amyloid-beta pathology: a link to Alzheimer's disease? Nat. Rev. Neurosci. 11, 361–370. doi: 10.1038/nrn2808, 20216546 PMC3979339

[ref29] Kang J. W. Vemuganti V. Kuehn J. F. Ulland T. K. Rey F. E. Bendlin B. B. (2024). Gut microbial metabolism in Alzheimer's disease and related dementias. Neurotherapeutics 21:e00470. doi: 10.1016/j.neurot.2024.e00470, 39462700 PMC11585892

[ref30] Kelly C. R. Khoruts A. Staley C. Sadowsky M. J. Abd M. Alani M. . (2016). Effect of fecal microbiota transplantation on recurrence in multiply recurrent *Clostridium difficile* infection: a randomized trial. Ann. Intern. Med. 165, 609–616. doi: 10.7326/M16-0271, 27547925 PMC5909820

[ref31] Klein S. L. Flanagan K. L. (2016). Sex differences in immune responses. Nat. Rev. Immunol. 16, 626–638. doi: 10.1038/nri.2016.90, 27546235

[ref32] Lin H. Peddada S. D. (2024). Multigroup analysis of compositions of microbiomes with covariate adjustments and repeated measures. Nat. Methods 21, 83–91. doi: 10.1038/s41592-023-02092-7, 38158428 PMC10776411

[ref33] Livingston G. Huntley J. Liu K. Y. Costafreda S. G. Selbaek G. Alladi S. . (2024). Dementia prevention, intervention, and care: 2024 report of the lancet standing commission. Lancet 404, 572–628. doi: 10.1016/S0140-6736(24)01296-0, 39096926

[ref34] Loane D. J. Pocivavsek A. Moussa C. E. Thompson R. Matsuoka Y. Faden A. I. . (2009). Amyloid precursor protein secretases as therapeutic targets for traumatic brain injury. Nat. Med. 15, 377–379. doi: 10.1038/nm.1940, 19287391 PMC2844765

[ref35] Luissint A. C. Parkos C. A. Nusrat A. (2016). Inflammation and the intestinal barrier: leukocyte-epithelial cell interactions, cell junction remodeling, and mucosal repair. Gastroenterology 151, 616–632. doi: 10.1053/j.gastro.2016.07.008, 27436072 PMC5317033

[ref36] Markle J. G. Frank D. N. Mortin-Toth S. Robertson C. E. Feazel L. M. Rolle-Kampczyk U. . (2013). Sex differences in the gut microbiome drive hormone-dependent regulation of autoimmunity. Science 339, 1084–1088. doi: 10.1126/science.1233521, 23328391

[ref37] McDonald D. Jiang Y. Balaban M. Cantrell K. Zhu Q. Gonzalez A. . (2024). Greengenes2 unifies microbial data in a single reference tree. Nat. Biotechnol. 42, 715–718. doi: 10.1038/s41587-023-01845-1, 37500913 PMC10818020

[ref38] Nicholson S. E. Watts L. T. Burmeister D. M. Merrill D. Scroggins S. Zou Y. . (2019). Moderate traumatic brain injury alters the gastrointestinal microbiome in a time-dependent manner. Shock 52, 240–248. doi: 10.1097/SHK.0000000000001211, 29953417

[ref39] O'Callaghan A. van Sinderen D. (2016). Bifidobacteria and their role as members of the human gut microbiota. Front. Microbiol. 7:925. doi: 10.3389/fmicb.2016.00925, 27379055 PMC4908950

[ref40] Opeyemi O. M. Rogers M. B. Firek B. A. Janesko-Feldman K. Vagni V. Mullett S. J. . (2021). Sustained dysbiosis and decreased fecal short-chain fatty acids after traumatic brain injury and impact on neurologic outcome. J. Neurotrauma 38, 2610–2621. doi: 10.1089/neu.2020.7506, 33957773 PMC8403202

[ref41] Org E. Mehrabian M. Parks B. W. Shipkova P. Liu X. Drake T. A. . (2016). Sex differences and hormonal effects on gut microbiota composition in mice. Gut Microbes 7, 313–322. doi: 10.1080/19490976.2016.1203502, 27355107 PMC4988450

[ref42] Parada Venegas D. la De Fuente M. K. Landskron G. Gonzalez M. J. Quera R. Dijkstra G. . (2019). Corrigendum: short chain fatty acids (SCFAs)-mediated gut epithelial and immune regulation and its relevance for inflammatory bowel diseases. Front. Immunol. 10:1486. doi: 10.3389/fimmu.2019.01486, 31316522 PMC6611342

[ref43] Pasam T. Dandekar M. P. (2023). Fecal microbiota transplantation unveils sex-specific differences in a controlled cortical impact injury mouse model. Front. Microbiol. 14:1336537. doi: 10.3389/fmicb.2023.1336537, 38410824 PMC10894955

[ref44] Pasam T. Dandekar M. P. (2024). Insights from rodent models for improving bench-to-bedside translation in traumatic brain injury. Methods Mol. Biol. 2761, 599–622. doi: 10.1007/978-1-0716-3662-6_40, 38427264

[ref45] Perez-Nievas B. G. Stein T. D. Tai H. C. Dols-Icardo O. Scotton T. C. Barroeta-Espar I. . (2013). Dissecting phenotypic traits linked to human resilience to Alzheimer's pathology. Brain 136, 2510–2526. doi: 10.1093/brain/awt171, 23824488 PMC3722351

[ref46] Plassman B. L. Havlik R. J. Steffens D. C. Helms M. J. Newman T. N. Drosdick D. . (2000). Documented head injury in early adulthood and risk of Alzheimer's disease and other dementias. Neurology 55, 1158–1166. doi: 10.1212/wnl.55.8.1158, 11071494

[ref47] Popescu C. Munteanu C. Anghelescu A. Ciobanu V. Spinu A. Andone I. . (2024). Novelties on Neuroinflammation in Alzheimer's disease-focus on gut and Oral microbiota involvement. Int. J. Mol. Sci. 25. doi: 10.3390/ijms252011272, 39457054 PMC11508522

[ref48] Pu D. Yao Y. Zhou C. Liu R. Wang Z. Liu Y. . (2024). FMT rescues mice from DSS-induced colitis in a STING-dependent manner. Gut Microbes 16:2397879. doi: 10.1080/19490976.2024.2397879, 39324491 PMC11441074

[ref49] Ramlackhansingh A. F. Brooks D. J. Greenwood R. J. Bose S. K. Turkheimer F. E. Kinnunen K. M. . (2011). Inflammation after trauma: microglial activation and traumatic brain injury. Ann. Neurol. 70, 374–383. doi: 10.1002/ana.22455, 21710619

[ref50] Ramos-Cejudo J. Wisniewski T. Marmar C. Zetterberg H. Blennow K. de Leon M. J. . (2018). Traumatic brain injury and Alzheimer's disease: the cerebrovascular link. EBioMedicine 28, 21–30. doi: 10.1016/j.ebiom.2018.01.021, 29396300 PMC5835563

[ref51] Serrano-Pozo A. Frosch M. P. Masliah E. Hyman B. T. (2011). Neuropathological alterations in Alzheimer disease. Cold Spring Harb. Perspect. Med. 1:a006189. doi: 10.1101/cshperspect.a006189, 22229116 PMC3234452

[ref52] Sil A. Erfani A. Lamb N. Copland R. Riedel G. Platt B. (2022). Sex differences in behavior and molecular pathology in the 5XFAD model. J Alzheimer's Dis 85, 755–778. doi: 10.3233/JAD-210523, 34864660

[ref53] Sivandzade F. Alqahtani F. Cucullo L. (2020). Traumatic brain injury and blood-brain barrier (BBB): underlying pathophysiological mechanisms and the influence of cigarette smoking as a premorbid condition. Int. J. Mol. Sci. 21:2721. doi: 10.3390/ijms21082721, 32295258 PMC7215684

[ref54] Soriano S. Curry K. Wang Q. Chow E. Treangen T. J. Villapol S. (2022). Fecal microbiota transplantation derived from Alzheimer's disease mice worsens brain trauma outcomes in wild-type controls. Int. J. Mol. Sci. 23:4476. doi: 10.3390/ijms23094476, 35562867 PMC9103830

[ref55] Stone J. R. Okonkwo D. O. Singleton R. H. Mutlu L. K. Helm G. A. Povlishock J. T. (2002). Caspase-3-mediated cleavage of amyloid precursor protein and formation of amyloid Beta peptide in traumatic axonal injury. J. Neurotrauma 19, 601–614. doi: 10.1089/089771502753754073, 12042095

[ref56] Sun J. Xu J. Ling Y. Wang F. Gong T. Yang C. . (2019). Fecal microbiota transplantation alleviated Alzheimer's disease-like pathogenesis in APP/PS1 transgenic mice. Transl. Psychiatry 9:189. doi: 10.1038/s41398-019-0525-3, 31383855 PMC6683152

[ref57] Tabassum S. Wu S. Lee C. H. Yang B. S. K. Gusdon A. M. Choi H. A. . (2025). Mitochondrial-targeted therapies in traumatic brain injury: from bench to bedside. Neurotherapeutics 22:e00515. doi: 10.1016/j.neurot.2024.e00515, 39721917 PMC11840356

[ref58] Tang X. Huang L. Ma W. Huang M. Zeng Z. Yu Y. . (2025). Intestinal 8 gingerol attenuates TBI-induced neuroinflammation by inhibiting microglia NLRP3 inflammasome activation in a PINK1/Parkin-dependent manner. Phytomedicine 140:156580. doi: 10.1016/j.phymed.2025.156580, 40058316

[ref59] Tang-Schomer M. D. Johnson V. E. Baas P. W. Stewart W. Smith D. H. (2012). Partial interruption of axonal transport due to microtubule breakage accounts for the formation of periodic varicosities after traumatic axonal injury. Exp. Neurol. 233, 364–372. doi: 10.1016/j.expneurol.2011.10.030, 22079153 PMC3979336

[ref60] Thal D. R. Rub U. Orantes M. Braak H. (2002). Phases of a beta-deposition in the human brain and its relevance for the development of AD. Neurology 58, 1791–1800. doi: 10.1212/wnl.58.12.1791, 12084879

[ref61] Treangen T. J. Wagner J. Burns M. P. Villapol S. (2018). Traumatic brain injury in mice induces acute bacterial Dysbiosis within the fecal microbiome. Front. Immunol. 9:2757. doi: 10.3389/fimmu.2018.02757, 30546361 PMC6278748

[ref62] Uryu K. Laurer H. McIntosh T. Pratico D. Martinez D. Leight S. . (2002). Repetitive mild brain trauma accelerates Abeta deposition, lipid peroxidation, and cognitive impairment in a transgenic mouse model of Alzheimer amyloidosis. J. Neurosci. 22, 446–454. doi: 10.1523/JNEUROSCI.22-02-00446.2002, 11784789 PMC6758680

[ref63] Vendrik K. E. W. Ooijevaar R. E. de Jong P. R. C. Laman J. D. van Oosten B. W. van Hilten J. J. . (2020). Fecal microbiota transplantation in neurological disorders. Front. Cell. Infect. Microbiol. 10:98. doi: 10.3389/fcimb.2020.00098, 32266160 PMC7105733

[ref64] Viejo L. Noori A. Merrill E. Das S. Hyman B. T. Serrano-Pozo A. (2022). Systematic review of human post-mortem immunohistochemical studies and bioinformatics analyses unveil the complexity of astrocyte reaction in Alzheimer's disease. Neuropathol. Appl. Neurobiol. 48:e12753. doi: 10.1111/nan.12753, 34297416 PMC8766893

[ref65] Vila-Castelar C. Akinci M. Palpatzis E. Aguilar-Dominguez P. Operto G. Kollmorgen G. . (2025). Sex/gender effects of glial reactivity on preclinical Alzheimer's disease pathology. Mol. Psychiatry 30, 1430–1439. doi: 10.1038/s41380-024-02753-9, 39384963 PMC11919761

[ref66] Villa A. Gelosa P. Castiglioni L. Cimino M. Rizzi N. Pepe G. . (2018). Sex-specific features of microglia from adult mice. Cell Rep. 23, 3501–3511. doi: 10.1016/j.celrep.2018.05.048, 29924994 PMC6024879

[ref67] Villapol S. Balarezo M. G. Affram K. Saavedra J. M. Symes A. J. (2015). Neurorestoration after traumatic brain injury through angiotensin II receptor blockage. Brain 138, 3299–3315. doi: 10.1093/brain/awv172, 26115674 PMC4731413

[ref68] Villapol S. Byrnes K. R. Symes A. J. (2014). Temporal dynamics of cerebral blood flow, cortical damage, apoptosis, astrocyte-vasculature interaction and astrogliosis in the pericontusional region after traumatic brain injury. Front. Neurol. 5:82. doi: 10.3389/fneur.2014.00082, 24926283 PMC4044679

[ref69] Villapol S. Loane D. J. Burns M. P. (2017). Sexual dimorphism in the inflammatory response to traumatic brain injury. Glia 65, 1423–1438. doi: 10.1002/glia.23171, 28608978 PMC5609840

[ref70] Villapol S. Yaszemski A. K. Logan T. T. Sanchez-Lemus E. Saavedra J. M. Symes A. J. (2012). Candesartan, an angiotensin II AT(1)-receptor blocker and PPAR-gamma agonist, reduces lesion volume and improves motor and memory function after traumatic brain injury in mice. Neuropsychopharmacology 37, 2817–2829. doi: 10.1038/npp.2012.152, 22892395 PMC3499714

[ref71] Vogt N. M. Kerby R. L. Dill-McFarland K. A. Harding S. J. Merluzzi A. P. Johnson S. C. . (2017). Gut microbiome alterations in Alzheimer's disease. Sci. Rep. 7:13537. doi: 10.1038/s41598-017-13601-y, 29051531 PMC5648830

[ref72] Wang J. Z. Grundke-Iqbal I. Iqbal K. (2007). Kinases and phosphatases and tau sites involved in Alzheimer neurofibrillary degeneration. Eur. J. Neurosci. 25, 59–68. doi: 10.1111/j.1460-9568.2006.05226.x, 17241267 PMC3191918

[ref73] Weisburg W. G. Barns S. M. Pelletier D. A. Lane D. J. (1991). 16S ribosomal DNA amplification for phylogenetic study. J. Bacteriol. 173, 697–703. doi: 10.1128/jb.173.2.697-703.1991, 1987160 PMC207061

[ref74] Wu Z. Wang Z. H. Liu X. Zhang Z. Gu X. Yu S. P. . (2020). Traumatic brain injury triggers APP and tau cleavage by delta-secretase, mediating Alzheimer's disease pathology. Prog. Neurobiol. 185:101730. doi: 10.1016/j.pneurobio.2019.101730, 31778772

[ref75] Xu G. Fromholt S. E. Chakrabarty P. Zhu F. Liu X. Pace M. C. . (2020). Diversity in aβ deposit morphology and secondary proteome insolubility across models of Alzheimer-type amyloidosis. Acta Neuropathol. Commun. 8:43. doi: 10.1186/s40478-020-00911-y, 32252825 PMC7137436

[ref76] Zhan X. Stamova B. Sharp F. R. (2018). Lipopolysaccharide associates with amyloid plaques, neurons and oligodendrocytes in Alzheimer's disease brain: a review. Front. Aging Neurosci. 10:42. doi: 10.3389/fnagi.2018.00042, 29520228 PMC5827158

[ref77] Zhao X. Zeng W. Xu H. Sun Z. Hu Y. Peng B. . (2023). A microtubule stabilizer ameliorates protein pathogenesis and neurodegeneration in mouse models of repetitive traumatic brain injury. Sci. Transl. Med. 15:eabo6889. doi: 10.1126/scitranslmed.abo6889, 37703352 PMC10787216

[ref78] Zhu D. Montagne A. Zhao Z. (2021). Alzheimer's pathogenic mechanisms and underlying sex difference. Cell. Mol. Life Sci. 78, 4907–4920. doi: 10.1007/s00018-021-03830-w, 33844047 PMC8720296

